# Comparative analysis of the soil microbiome and carbohydrate content of *Anthoxanthum nitens* (Sweetgrass) and other Poaceae grass tissues and associated soils

**DOI:** 10.3389/fmicb.2024.1384204

**Published:** 2025-04-28

**Authors:** Marissa L. King, Barinder Bajwa, Naomi Hanna, Xiaohui Xing, Kristin E. Low, Patrick Neuberger, Erin Hall, Michael Veltri, Brett Weighill, Leeann Klassen, Noreen Plain Eagle, William Big Bull, Laura S. Lynes, Tony Montina, Philippe J. Thomas, Monika A. Gorzelak, D. Wade Abbott

**Affiliations:** ^1^Lethbridge Research and Development Centre, Agriculture and Agri-Food Canada, Lethbridge, AB, Canada; ^2^Department of Chemistry and Biochemistry, University of Lethbridge, Lethbridge, AB, Canada; ^3^Department of Geography and Environment, University of Lethbridge, Lethbridge, AB, Canada; ^4^Peigan Board of Education, Piikani Nation, Brocket, AB, Canada; ^5^Piikani Nation Lands Department, Piikani Nation, Brocket, AB, Canada; ^6^The Resilience Institute, Canmore, AB, Canada; ^7^Environment and Climate Change Canada, National Wildlife Research Centre, Ottawa, ON, Canada

**Keywords:** Sweetgrass, *Anthoxanthum nitens*, soil microbiome, glycomics, Indigenous knowledge, transdisciplinary

## Abstract

Sweetgrass (*Anthoxanthum nitens*) is a culturally and environmentally significant perennial grass to many Indigenous Peoples; however, little is known about the potential of Sweetgrass as a contributor to soil health, biodiversity, and climate adaptation. Here, a team of transdisciplinary experts from academia, a non-governmental organization, and a First Nation community collaborated to investigate the structural composition of the rhizomes, stems, and leaves of greenhouse-grown Sweetgrass in comparison to other Poaceae grass members found in a nearby field. The data shows that the monosaccharide composition of *A. nitens* was evenly distributed throughout the three tissues, and that cellulose was the predominant polysaccharide followed by glucuronoararbinoxylans. There were lesser amounts of xyloglucans, mixed-linkage glucans, homogalacturonans, and rhamnogalacturonans as the hemicellulosic and pectic polysaccharides, respectively. The carbohydrate composition seen in *A. nitens* was consistent with the other Poaceae grasses evaluated in this study, with the exception of *Setaria chondrachne*, which contained elevated pectin levels in its stems and leaves. Additionally, the analysis of the carbohydrate content within the soil samples revealed a higher abundance of carbohydrates within greenhouse soil when compared to field soil samples, with significantly more mannose, galactose, and galacturonic acid. Further, there were structural differences in the microbial communities across sampling sites, including a significant increase in the abundance of *Bacillus* spp. in the greenhouse soil. Overall, this study provides the glycome and associated soil microbial community baseline for greenhouse-grown Sweetgrass.

## 1 Introduction

*Anthoxanthum nitens* (Weber) Y. Schouten & Veldkamp (aka., *Hierochloe odorata* (L.) P. Beauv), commonly known as Sweetgrass, is a perennial Poaceae grass found throughout North America, Europe, and Asia. In Canada, Sweetgrass is considered a culturally and environmentally significant plant for First Nations, Inuit and Métis peoples and is commonly used for ceremonial protocol, medicine, as a material for weaving baskets or other crafts (Moerman, 1998; Kavasch and Baar, 1999; Shebitz and Kimmerer, 2005), or as a parable by Elders (Brant, 2022). Sweetgrass, especially the roots, are scented with a vanilla-like fragrance that is produced by coumarin (Ueyama et al., 1991). This volatile compound along with its coumadin and warfarin derivates have demonstrated anticoagulant activity (Sharifi-Rad et al., 2021). Additionally, coumarin and its 5,8-dihydroxycoumarin and 5-hydroxy-8-O-β-d-glucopyranosycoumarin derivatives have also been explored for their antioxidant properties. Sweetgrass extracts have previously shown a decrease in lipid oxidation in *in vitro* assays (Pukalskas et al., 2002) and rats administered ethanol (Dobrzyńska et al., 2013).

While the volatile and antioxidant properties of Sweetgrass have been well-documented, the carbohydrate profile, or glycome, of this plant remains relatively unknown. Sweetgrass has been previously analyzed for its fiber content in the search for sustainable materials to reinforce polymeric composites, revealing contents of 70.4% cellulose, 21.5% hemicellulose, and 8.1% lignin (Dalmis et al., 2020). However, this study did not investigate the polysaccharide profile within the plant and differences between its tissues. Using glycomics to profile the carbohydrate content of Sweetgrass, the potential Sweetgrass has for carbon sequestration can start to be revealed, as this rhizomatous grass stores carbon in its extensive rhizome network within soil (Winslow, 2000; Panchal et al., 2022). Sweetgrass has an expansive habitat much of which is vulnerable to the impacts of climate change, especially drought in prairie regions (Schneider, 2013). Thus, evaluating the carbon sequestration potential of Sweetgrass carbohydrates and the impact of a decline in abundance (Shebitz and Kimmerer, 2004) are important considerations.

The flow of carbohydrates within and between different ecosystems is deeply dependent on the composition of the soil microbial communities within each environment (Low et al., 2023). Plants interact with the soil microbial community through the production of root exudates, mucilage, and other secretions (Jones et al., 2009). Root exudates are primarily composed of carbohydrates, along with amino acids, organic acids, fatty acids, and secondary metabolites (Bais et al., 2006; Jones et al., 2009; Vranova et al., 2013). Exudate carbohydrates are quickly utilized by bacteria and fungi, which contributes to the assembly of the soil microbial community (Zhalnina et al., 2018). Depending on the exudate composition, plants can modify the soil through changing the soil pH, attracting beneficial microbes, and chelating or releasing toxic compounds (Vives-Peris et al., 2020). In addition to rhizodeposition, carbohydrates can also enter the soil through manure or compost, or as plant cell wall biomass in litter, where decomposition by soil microorganisms influences the turnover of soil organic matter (Schimel and Schaeffer, 2012). Unlike root exudates, recalcitrant cell wall polysaccharides require complex enzymatic pathways produced by capable microorganisms to be digested (Pérez et al., 2002). Overall, carbohydrates stemming from root exudates or cell wall litter not only mediate interaction between plants and soil microbiota, but also affect interactions between microbial members of the soil community (Sasse et al., 2018).

This study was conducted in partnership with the Piikani Nation, a proud First Nation community in southern Alberta, Canada and member of the Blackfoot Confederacy; the Resilience Institute, a climate-focused charity with a long-standing relationship with the Piikani Nation; two federal government departments: Environment and Climate Change Canada and Agriculture and Agri-Food Canada; and the University of Lethbridge. The Piikani Nation has ~4,200 registered members, where an estimated 40% of the members live off reserve in urban centers that surround Nation lands. They occupy two reserves; the main reserve along Alberta Highway No. 3 covers an area of 42,6127 hectares (105,200 acres) and the timber reserve (No. 147B) located in the Porcupine Hills to the northwest corner covers an area of 2,979 hectares (7,360 acres). Similar to many Indigenous communities, the Piikani Nation commonly uses Sweetgrass for cultural purposes, but it is becoming harder to find on the land (Plain Eagle, 2023). Here, the monosaccharide composition of the rhizome, stem, and leaf fractions of greenhouse-grown Sweetgrass was determined and compared to other Poaceae grass members collected from a nearby field on Piikani Nation lands. The chemical structures of polysaccharides were investigated using glycosidic linkage analysis by gas chromatography-mass spectrometry (GC-MS) and gas chromatography-flame ionization detection (GC-FID) analyses. Additionally, greenhouse and field soils associated with each plant were assessed for their chemical and carbohydrate content. Lastly, greenhouse and field soil samples were assessed using 16S rRNA gene sequencing to determine the bacterial composition of the soil microbial communities as a function of their habitats and growing conditions.

## 2 Materials and methods

### 2.1 Collection of grass and soil samples

Prior to sample collection, the Piikani Nation and their community partners at the Resilience Institute, hosted a Sweetgrass Partnership Ceremony (September 9, 2022, Piikani Reserve 147 and 147B). Scientists from the University of Lethbridge and Agriculture and Agri-Food Canada, the president of the Resilience Institute, and invited guests joined Elders and other knowledge holders from the Piikani Nation for the ceremony. Elder Peter Strikes with a Gun gave a blessing for the collaborative work and expressed how Sweetgrass represented a symbol not just of their history, but of their future (Strikes with a Gun, 2022). Following the ceremony, a traditional Blackfoot meal was provided consisting of Saskatoon berry soup and fry bread. This Partnership Ceremony was an essential part of the research method for this study and paved the way for the collection of samples.

Guided by Piikani knowledge holders and students, six grass samples were collected from a 1 m^2^ nursery bed located within a greenhouse next to the Piikani Nation Secondary School (Brocket, AB, Canada; 49.54332°N, 113.75018°W) on October 4, 2022. Additionally, six grass samples were collected from a 5 m^2^ section within a field on the main Piikani Reserve where Sweetgrass used to be commonly gathered (Brocket, AB, Canada; 49.54925°N, 113.73252°W; October 4, 2022). All plant samples were dug out and divided into leaf; stem; and rhizome tissues; each tissue sample was immediately frozen on dry ice and stored at −80°C. Soil and plant samples were freeze-dried and ball-milled to a fine powder using a Mixer Mill MM400 (Retsch, Germany), and stored at room temperature until processed further.

Soil samples associated with the plants collected from the greenhouse (*n* = 6) and field (*n* = 6) sampling sites, were also collected from the top layer (0–15 cm), and immediately frozen on dry ice and stored at −80°C until further processing. Soil samples were then sieved (2 mm) before being divided into two parts: one for 16S rRNA gene sequencing and microbial community analysis, and the second for glycomics. Additionally, six soil samples were collected from a “baseline” sampling site located adjacent to the greenhouse, which was the site originally used to collect soil for use in the greenhouse that was supplemented with bloodmeal as a fertilizer. Baseline soil samples were treated similarly to the greenhouse and field soils, except baseline soil samples were only studied using 16S rRNA gene sequencing.

### 2.2 Identification of grass species through ITS sequencing

To identify the grass species that were collected from the greenhouse and field sites, the internal transcribed spacer 1 (ITS1) region was used for DNA barcoding. DNA was extracted from all greenhouse leaf samples (50 mg) using the DNeasy Plant Pro kit (Qiagen, Canada) following the manufacturer's instructions. The ITS1 region was targeted for amplification by PCR using the primers: ITS-p5 (CCTTATCAYTTAGAGGAAGGAG) and ITS-p4 (CCGCTTAKTGATATGCTTAAA) (Cheng et al., 2016). Amplification was performed using iProof™ High-Fidelity DNA polymerase (Bio-Rad, Canada) with PCR cycles as follows: initial denaturation step of 98°C for 1 min before 16 cycles of: 98°C for 10 s, 58–0.5°C cycle^−1^ for 20 s, and 72°C for 1 min, before 30 cycles of: 98°C for 10 s, 52°C for 20 s, and 72°C for 1 min, with a final extension at 72°C for 5 min. PCR products (~750 bp) were sent for Sanger sequencing (Eurofins Genomics, Canada), and sequencing results were paired using Sequencher (version 5.4.6) and run through NCBI's nucleotide BLAST suite (Altschul et al., 1990).

### 2.3 Preparation of alcohol insoluble residue

Alcohol insoluble residue (AIR) of rhizome, stem, and leaf sections of the greenhouse and field grass samples, along with the soil collected from each collection site, were prepared according to the literature (Pattathil et al., 2012) with modifications (Wood et al., 2018; Klassen et al., 2023). Briefly, 100 mg of each ball-milled plant sample and 10 g of each ball-milled soil sample was suspended in 40 mL of 80% (v/v) ethanol and placed on a rotator for 8 h, for a total of three washes, followed by a single wash of acetone and methanol, each for 20 min. Samples were centrifuged (3,000 × *g*, 30 min) between solvent washes and the supernatant was discarded. The residue after the final wash was vacuum-dried by SpeedVac (Thermo Scientific, USA).

### 2.4 Preparation of partially methylated alditol acetates from plant cell walls

Cell wall residue from AIR was de-starched by incubation with α-amylase (E-BLAAM, Megazyme) in 100 mM maleic acid buffer (pH 6.0) containing 100 mM NaCl and 3 mM CaCl_2_ for 8 h at 70°C followed by incubation with amyloglucosidase (E-AMGDFPD, Megazyme) for 4 h at 50°C. The de-starched samples were extensively dialyzed (MWCO 3.5 kDa) against deionized water and then freeze-dried. For linkage analysis of neutral sugars, 5 mg of each AIR was permethylated by 0.6 mL of methyl iodide in 1 mL of dimethyl sulfoxide (DMSO) with the presence of excess amount of sodium hydroxide (NaOH) (Jones et al., 2020; Low et al., 2020; Badhan et al., 2022). The methylation product was cleaned up by partition between dichloromethane and 10% (v/v) acetic acid over ice one time and then with deionized water three times. The final lower phase was evaporated to dryness, and then the methylation process was repeated two more times. The per-*O*-methylated samples were subsequently hydrolyzed to monosaccharides with 2 mL of 4 M trifluoroacetic acid (TFA) for 4 h at 100°C, reduced with 10 mg of sodium borodeuteride (NaBD_4_, 99 atom % D, Alfa Aesar) dissolved in 1 mL of deionized water for 16 h at room temperature (Low et al., 2020), and then per-acetylated with 2.5 mL of mixture of acetic anhydride and TFA (5:1, v/v) at 60°C for 1 h (Voiges et al., 2012; Robb et al., 2022; Chang et al., 2023). The resulting partially methylated alditol acetates (PMAAs) were redissolved in 3 mL of dichloromethane, and washed with saturated deionized water solution of sodium bicarbonate then deionized water three times. After that, the lower phase was passed through a glass wool-plugged Pasteur pipette loaded with anhydrous sodium sulfate powder (Yu et al., 2017), followed by evaporation to dryness and redissolved in ethyl acetate for GC-MS analysis. In a separate experiment, uronic acids in approximately 5 mg of dry AIR powder were carboxyl reduced to 6,6-dideuterated neutral sugars by NaBD_4_ reduction of their methyl esters generated by weak methanolysis (0.5 M methanolic HCl, 80°C, 20 min) (Chong et al., 2019; Hosain et al., 2019; Muhidinov et al., 2020), followed by converting to PMAAs using the same procedure as described above. Two technical replicates were conducted for each sample.

### 2.5 GC-MS analysis of PMAAs

All PMAA samples were tested on an Agilent 7890A-5977B GC-MS system (Agilent Technologies, Santa Clara, CA) installed with a medium-polarity Supelco SP-2380 column (60 m × 0.25 mm × 0.2 μm; Sigma-Aldrich, USA) with a constant flow rate of helium (0.8 mL min^−1^). Samples were injected at an inlet temperature of 250°C with a 10:1 split ratio. The oven temperature was programmed to start at 120°C (hold 1 min) followed by increasing at 3°C min^−1^ to 200°C (hold 50 min) then 3°C min^−1^ to 250°C (hold 20 min). The EI-MS spectra of the PMAAs were interpreted by comparing them with those of reference derivatives and by referring to the literature (Carpita and Shea, 1989). The glycosidic linkage composition (mol%) was calculated following the published protocol (Pettolino et al., 2012). Visualization of the plant glycosidic linkage data was carried out using R (version 4.2.2) in R-studio (version 2022.02.3 Build 492) with the packages: ggplot2 (Wickham, 2016) and reshape2 (Wickham, 2007). Correlations amongst the glycosidic linkages present within the rhizome, stem, and leaf tissues of the greenhouse and field plants were analyzed with principal component analysis (PCA) plots using R in R-studio with the packages: dplyr (Wickham et al., 2023), forcats (Wickham, 2023), and ggplot2 (Wickham, 2016). Polysaccharide compositions were estimated from the linkage data (Supplementary Tables S3, S4) by adapting the published calculations from Pettolino et al. (2012).

### 2.6 Analysis of total carbohydrate content and monosaccharide composition of soil samples

In experimental duplicates, ball-milled air powder of soil (250 mg) was hydrolyzed by magnetic stirring in 1 mL of 12 M H_2_SO_4_ for 2 h, followed by adding 11 mL of deionized water to dilute the acid and then incubation at 100°C for 2 h with magnetic stirring. The hydrolysate was transferred to a 50 mL tube, along with 12 mL of deionized water used to wash the original tube for hydrolysis. Samples were centrifuged three times (3,000 × *g*, 10 min), where between spins the resulting supernatants were pooled with three deionized water washes of the pellet. 5 M NaOH solution (2 mL) was added to neutralize each supernatant. The resulting hydrolysates of soil samples were filtered (0.2 μm) for monosaccharide analysis using high-performance anion-exchange chromatography with pulsed amperometric detection (HPAEC-PAD), while hydrolysates were not filtered for the soil total carbohydrate assay.

The total carbohydrate contents of the soil AIRs were determined calorimetrically using the phenol-sulphuric acid assay (Dubois et al., 1956). Briefly, 2 mL of the above-mentioned hydrolysate was mixed with 50 μL of 80% phenol water solution, immediately followed by adding 3 mL of 18 M H_2_SO_4_. The solutions were vortexed and then cooled to room temperature followed by reading the absorbance at 490 nm with a Varian Cary 300 UV-Vis spectrophotometer (Agilent Technologies, Santa Clara, CA) against a reagent blank prepared from 2 mL of deionized water instead of the hydrolysate. The carbohydrate content of each soil sample was measured against a calibration curve prepared from 20 to 60 μg mL^−1^ of glucose solution. Total carbohydrate content was calculated based on the soil dry weight, where the mean ± standard deviation (*n* = 2) was used to plot the data in GraphPad Prism (version 8.0.2).

Absolute monosaccharide quantification was performed using HPAEC-PAD on all greenhouse and field soil samples. HPAEC-PAD was performed with a Dionex ICS-3000 chromatography system (Thermo Scientific) equipped with an autosampler and a pulsed amperometric (PAD) detector. 10 μL of each hydrolyzed sample was injected onto an analytical (3 × 150 mm) CarboPac PA20 column (Thermo Scientific) with a CarboPac PA20 guard column (Thermo Scientific). Neutral and uronic acid sugar samples were eluted at a flow rate of 0.3 mL min^−1^ with a background of NaOH (buffer A), and a gradient of sodium acetate (NaOAc; buffer B) (Supplementary Table S1) at 15°C, where amino sugars were eluted similarly but with a different gradient (Supplementary Table S2). The elution of each run was monitored with a PAD detector (standard quadratic waveform). For quantification of neutral and uronics, a mixture of monosaccharides was used in a known concentration series with the following concentrations for a 1X solution with 99 μM maltotriose used as an internal standard (ISTD): 119 μM fucose (Fuc), 132 μM arabinose (Ara), 132 μM ribose (Rib), 110 μM galactose (Gal), 110 μM glucose (Glc), 110 μM mannose (Man), 132 μM xylose (Xyl), 121 μM rhamnose (Rha), 110 μM glucosamine (GlcN), 101 μM guluronic acid (GulA), 98 μM galacturonic acid (GalA), 101 μM mannuronic acid (ManA), and 101 μM glucuronic acid (GlcA). For amino sugar quantification, a mixture of monosaccharides was used in a known concentration series with the following concentrations for a 1X solution with 99 μM maltotriose used as an ISTD: 112 μM GlcN, 112 μM galactosamine (GalN), and 112 μM mannosamine (ManN). Monosaccharide quantification was performed using Chromeleon software (Thermo Scientific) from triplicate HPAEC-PAD injections. The mean ± standard deviation (*n* = 3) was used to plot the data in GraphPad Prism (version 8.0.2). To compare differences in the amount of monosaccharides in the greenhouse and field soils, a two-way analysis of variance (ANOVA) test using a false discovery approach was conducted using GraphPad Prism (version 8.0.2).

### 2.7 16S rRNA gene sequencing of soil microbial communities

DNA was extracted from all baseline, field, and greenhouse rhizosphere soil samples (250 mg, 2 mm sieved) using the PowerSoil Pro kit (Qiagen, Canada) following the manufacturer's instructions. Purified DNA samples were sent to Génome Québec (Montréal, QC, Canada) for Illumina MiSeq PE250 16S rRNA gene sequencing using the primers 515F (GTGCCAGCMGCCGCGGTAA) and 806R (GGACTACHVGGGTWTCTAAT) targeting the V4 region. A total of 908,770 paired end reads were quality trimmed using Trimmomatic (Bolger et al., 2014) with a sliding window of 5:20. Trimmed fasta files were merged and classified using Kraken 2 (Wood et al., 2019) according to the Silva 138 SSU database. Bracken (Lu et al., 2017) was used to estimate bacterial and archaeal abundance at the genus level. Community analysis, statistics, and plotting of the Bracken output files were performed using R (version 4.2.2) in R-studio (version 2022.02.3 Build 492) with the packages: phyloseq (McMurdie and Holmes, 2013), ggplot2 (Wickham, 2016), picante (Kembel et al., 2010), rioja (Juggins, 2022), and vegan (Oksanen et al., 2023).

Statistical analyses were carried out using normalized read abundances and classification at the genus level. Normalized read abundances were calculated with the “decostand()” function (method = total) from the vegan package (Oksanen et al., 2023). Non-metric multi-dimensional scaling (NMDS) analyses based on Bray-Curtis dissimilarity were performed using the vegdist() function of vegan (Oksanen et al., 2023), to visualize the separation between soil sampling sites. A permutational multivariant analysis of variance (PERMANOVA) was used to assess the effect of soil type on the compositions of rhizosphere-associated microbial communities, using the adonis2 function in vegan (Oksanen et al., 2023) with 999 permutations. Observed OTUs, Chao1, Shannon, and Simpson indices were calculated in R as a measure of community alpha diversity using the estimate richness function in the phyloseq package (McMurdie and Holmes, 2013). Changes in alpha diversity between the different sampling locations were assessed using the Kruskal-Wallis test with *post-hoc* Dunn's multiple comparison in GraphPad Prism (version 8.0.2).

### 2.8 Soil chemistry

Soils were very dry upon arrival in the lab and determined to contain 0% moisture using the thermogravimetric method and thus were only air-dried at room temperature for 24 h and ground to 2 mm. A subsample of each soil was fine ground to 60 μm. NO_3_-N and NH_4_-N were extracted from the 2 mm subsample using a 2.0 M KCl extraction at 25°C and measured with an Autoanalyzer 3 (EasyChem Pro, Systea Analytical Technology, Anagni, Italy). The 2 mm subsample was used to determine pH with a 2:1 soil slurry (water:soil) and an Orion Star 211 pH probe (ThermoFisher Scientific, USA). The 60 μm ground sample was analyzed for total carbon (TC) and total nitrogen (TN) using dry combustion with an Elemental analyzer (CE Instruments). Total organic carbon (TOC) was determined by acidification with 0.6 N HCl and inorganic carbon was calculated by determining the difference between TC and TOC. Changes in the soil physio-chemical data parameters were assessed in R-studio (version 2023.06.1 Build 524) using a Kruskal-Wallis test with a *post-hoc* two sided Dunn test, that included a Benjamani-Hochberg multiple comparisons correction factor from the FSA (version 0.9.5) package (Ogle et al., 2023).

## 3 Results

### 3.1 ITS sequencing

DNA barcoding using the ITS1 region was used to identify the grass species that were collected from the greenhouse and field sites. Based on the top BLAST hit, all six greenhouse samples were confirmed to be Sweetgrass (*A. nitens*, Table 1), whereas the field samples were identified as different grass species including blue wild rye (*Elymus glaucus*), Pumpelly's bromegrass (*Bromus pumpellianus*), couch grass (*Elymus repens*), and *Setaria chondrachne* (Table 1).

**Table 1:** Identification of grass species collected from the greenhouse and field sites within the Piikani Nation using ITS1 sequence similarity.

**Sample**	**Top BLAST hit**	**Percent identity (%)**
GH1	*Anthoxanthum nitens* voucher CMN:CAN	97.3
GH2	*Anthoxanthum nitens* voucher CMN:CAN	98.1
GH3	*Anthoxanthum nitens* voucher CMN:CAN	97.7
GH4	*Anthoxanthum nitens* voucher CMN:CAN	97.9
GH5	*Anthoxanthum nitens* voucher CMN:CAN	97.6
GH6	*Anthoxanthum nitens* voucher CMN:CAN	98.3
F1	*Elymus glaucus* strain QH-5	83.6
F2	*Bromus pumpellianus* voucher CAN:528978	96.3
F3	*Setaria chondrachne* voucher HCCN-PJ008548-PB-261	79.5
F4	*Elymus repens* isolate ER1	92.5
F5	*Elymus repens* isolate ER1	89.4
F6	*Elymus repens* isolate ER1	98.9

Top BLAST hit is displayed.

### 3.2 Glycosidic linkage composition of greenhouse-grown Sweetgrass rhizome, stem, and leaf sections

**Figure 1:**
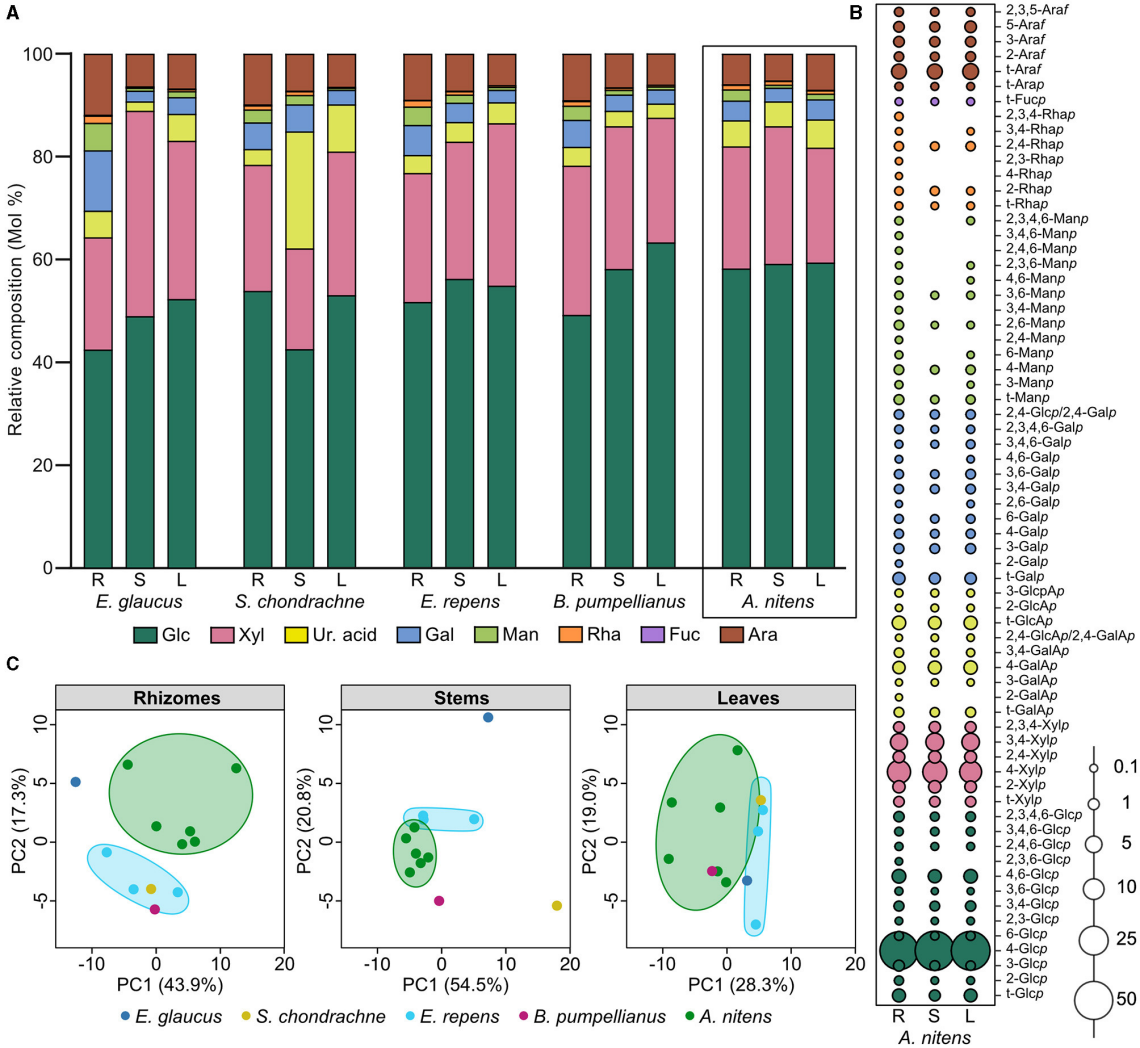
Carbohydrate content of greenhouse-grown Sweetgrass and other grass species. **(A)** Relative monosaccharide composition (mol%) of the cell walls present in the rhizome, stem, and leaf sections of greenhouse-grown Sweetgrass (*Anthoxanthum nitens*), and field grass species: *Elymus glaucus, Setaria chondrachne, Elymus repens*, and *Bromus pumpellianus*. **(B)** Relative composition (mol%) of glycosidic linkages present in the cell walls of greenhouse-grown Sweetgrass (*A. nitens*) rhizome, stem, and leaf sections. **(C)** Principal component analysis of glycosidic linkages obtained from the rhizome, stem, and leaf sections of each grass species; *E. glaucus* (dark blue), *S. chondrachne* (yellow), *E. repens* (light blue), and *B. pumpellianus* (fuchsia) collected from the field sampling site, and *A. nitens* (green) collected from the greenhouse.

The rhizomes, stems, and leaves of greenhouse-grown Sweetgrass tissues were assessed for their polysaccharide content through monosaccharide composition and glycosidic linkage analysis. The comparison of the individual monosaccharides based on relative abundances within the three Sweetgrass tissue sections, revealed a consistent monosaccharide distribution throughout the plant (Figure 1A; Table 2). Glucose (Glc) was the predominant monosaccharide (58.1–59.3%), followed by xylose (Xyl; 22.3–26.8%), arabinose (Ara; 5.3–7.1%), uronic acids (Ur. acid; 4.8–5.5%), galactose (Gal; 2.7–3.9%), mannose (Man; 0.6–2.2%), and trace amounts of rhamnose (Rha; 0.7–0.9%) and fucose (Fuc; 0.1%). The primary glucose linkages were 4-Glc*p*, 4,6-Glc*p*, and t-Glc*p*, whereby all three linkages were abundant in the three Sweetgrass tissues (*A. nitens;* Figure 1B; Supplementary Table S3). As the samples were de-starched prior to undergoing glycosidic linkage analysis, a majority of the 4-Glc*p* was presumed to stem from cellulose, which is consistent with the previous study (Dalmis et al., 2020), while the 4,6-Glc*p* and t-Glc*p* linkages likely accounted for xyloglucans and mixed-linkage glucans. The second most abundant polysaccharide found throughout Sweetgrass was determined to be glucuronoararbinoxylans (GAX) based on the presence and relative abundances of the 4-Xyl*p*, 3,4-Xyl*p*, 2,4-Xyl*p*, t-Ara*f* , and t-GlcA*p* linkages (Figure 1B; Supplementary Table S3) (Izydorczyk and Biliaderis, 1995; Smith and Harris, 1999). Additionally, the presence of various rhamnose linkages (i.e., t-Rha*p*, 2-Rha*p*, 4-Rha*p*, 2,3-Rha*p*, 2,4-Rha*p*, 3,4-Rha*p*, and 2,3,4-Rha*p*) is indicative of rhamnogalacturonans being present within Sweetgrass (Pettolino et al., 2012). However, these rhamnose linkages were sporadically present within the stem and leaf tissues of Sweetgrass, whereas all seven were present within the rhizomes (Figure 1B; Supplementary Table S3). In terms of uronic acid abundances within Sweetgrass, 4-GalA*p* and t-GalA*p* were present throughout the plant, therefore it was predicted that homogalacturonans (HGs) composed 1.4–4.1% of the total linkages in Sweetgrass.

**Table 2:** Relative monosaccharide composition (Mol %) calculated from glycosidic linkage compositions for rhizome (R), stems (S), and leaves (L) harvested from *A. nitens, E. glaucus, B. pumpellianus, S. chondrachne*, and *E. repens*.

**Monosaccharide**	***A. nitens***			***E. glaucus***			***B. pumpellianus***			***S. chondrachne***			***E. repens***		
	**R**	**S**	**L**	**R**	**S**	**L**	**R**	**S**	**L**	**R**	**S**	**L**	**R**	**S**	**L**
Ara	6.0	5.3	7.1	11.9	6.4	6.8	9.1	6.6	6.1	10.0	7.3	6.5	9.0	7.3	6.2
Fuc	0.1	0.1	0.1	0.2	0.0	0.1	0.2	0.1	0.0	0.1	0.1	0.0	0.1	0.1	0.0
Gal	3.8	2.7	3.9	11.8	2.0	3.2	5.2	3.2	2.7	5.1	5.2	2.8	5.8	3.8	2.4
Glc	58.1	59.0	59.3	42.3	48.7	51.9	49.2	57.9	62.8	53.8	42.5	52.8	51.4	56.1	54.6
Man	2.2	0.6	1.1	5.3	0.6	1.2	2.8	0.9	0.6	2.5	1.9	0.4	3.6	1.6	0.6
Rha	0.9	0.7	0.7	1.4	0.3	0.4	0.9	0.4	0.3	0.9	0.7	0.2	1.2	0.6	0.3
Xyl	23.7	26.8	22.3	21.8	39.7	30.6	29.0	27.6	24.1	24.5	19.6	27.8	24.9	26.6	31.4
GalA	2.6	2.7	3.1	3.1	0.4	3.1	1.3	1.0	0.9	1.2	16.1	6.3	1.4	1.8	2.3
GlcA	2.5	2.1	2.4	2.0	1.5	2.1	2.4	2.0	1.9	1.9	6.6	2.8	2.2	2.0	1.8

Two separate experiments were conducted for each sample.

### 3.3 Glycosidic linkage composition of the rhizome, stem, and leaf sections of other grass species collected from a nearby field

A higher degree of glycosidic diversity was seen between the three tissue sections of each field grass species. Interestingly, the stems of *S. chondrachne* contained the highest abundance of uronic acid (22.8%; Figure 1A; Table 2), mostly attributed to 4-GalA*p* and t-GlcA*p* whose abundances were increased within this section (Supplementary Figure S1). Additionally, *S. chondrachne* leaves also contained increased uronic acid levels (9.2%), with increased 4-GalA*p* and t-GlcA*p* but to a lesser extent than the stem section. Based on the abundances of these two uronic acid linkages, homogalacturonan is predicted to be more abundant within this field grass species. Specifically, based upon the proportion of 3,4-GalA*p* to 4-GalA*p* and the presence of rhamnose linkages (i.e., t-Rha*p*, 2,3- Rha*p*, 2,3,4- Rha*p*), where some of the GalA could be attributed to rhamnogalacturonan II (Pettolino et al., 2012). Further, the 4-ManA*p* linkage was only found in the stem and leaf tissues of *S. chondrachne* (Supplementary Figure S1). In contrast, the stem section of *E. glaucus* had the highest abundance of xylose (39.9%), whereas its rhizomes contained higher amounts of arabinose (11.9%), galactose (11.8%), mannose (5.3%), and rhamnose (1.4%) (Figure 1A; Table 2) in comparison to the other tissue sections of *E. glaucus*. Intriguingly, the leaves of *E. glaucus* had a similar neutral monosaccharide profile as the stems, and similar uronic acid content as the rhizomes. Increased levels of arabinose and galactose linkages such as t-Ara*p*, 6-Gal*p*, 3,6-Gal*p* are indicative of arabinogalactans, whereas the presence of mannose linkages (i.e., 4-Man*p*, 4,6-Man*p*) could be attributed to homomannan, glucomannan, or glucogalactomannan (Pettolino et al., 2012). With *E. repens* and *E. glaucus* being members of the same genus, the monosaccharide profiles of both showed comparable compositions. However, less dramatic proportions of xylose and galactose were seen in the *E. repens* stem and rhizome tissues, respectively (Figure 1A; Table 2). The stems and leaves of *B. pumpellianus* had similar profiles to each other, whereas the rhizomes of *B. pumpellianus* had increased levels of xylose (29.0%), arabinose (9.1%), galactose (5.2%), and mannose (2.8%) in comparison to its stem and leaf sections (Figure 1A; Table 2).

### 3.4 Comparison of glycosidic linkage compositions across plant sections and between different grass species

Amongst all grass species analyzed, the rhizome section of each plant contained an increased relative abundance of mannose and rhamnose (Figure 1A; Table 2), where a greater amount of diverse glycosidic linkages, such as 3,4-Rha*p*, 2-Rha*f* , 2,4,6-Man*p*, 4,6-Man*p*, 2,4-Man*p*, and 2-Gal*p*, were commonly found in the rhizomes but seldomly found in the other plant tissue sections of Sweetgrass and the field grasses (Supplementary Figure S1). The examination of the glycosidic linkage profiles of Sweetgrass and the field grasses revealed that the 2,4-GlcA*p*/2,4-GalA*p*, 3-GalA*p*, 2-Rha*p*, 2,3-Glc*p* linkages were consistently present in all three Sweetgrass tissues, but sporadically found in the field grass sections. Based on the linkage data for each plant tissue, PCA plotting revealed closely grouped data points belonging to the six greenhouse-grown Sweetgrass plants suggesting that these samples contained similar linkage compositions for their rhizome and stem sections (Figure 1C). The leaves of *B. pumpellianus* were aligned within the confidence interval of the leaves from Sweetgrass, indicating possible similarities between the two species. The most drastic difference in linkage compositions between greenhouse-grown Sweetgrass and the field grass species was seen in the rhizomes, where no overlap was observed (Figure 1C).

**Figure 2:**
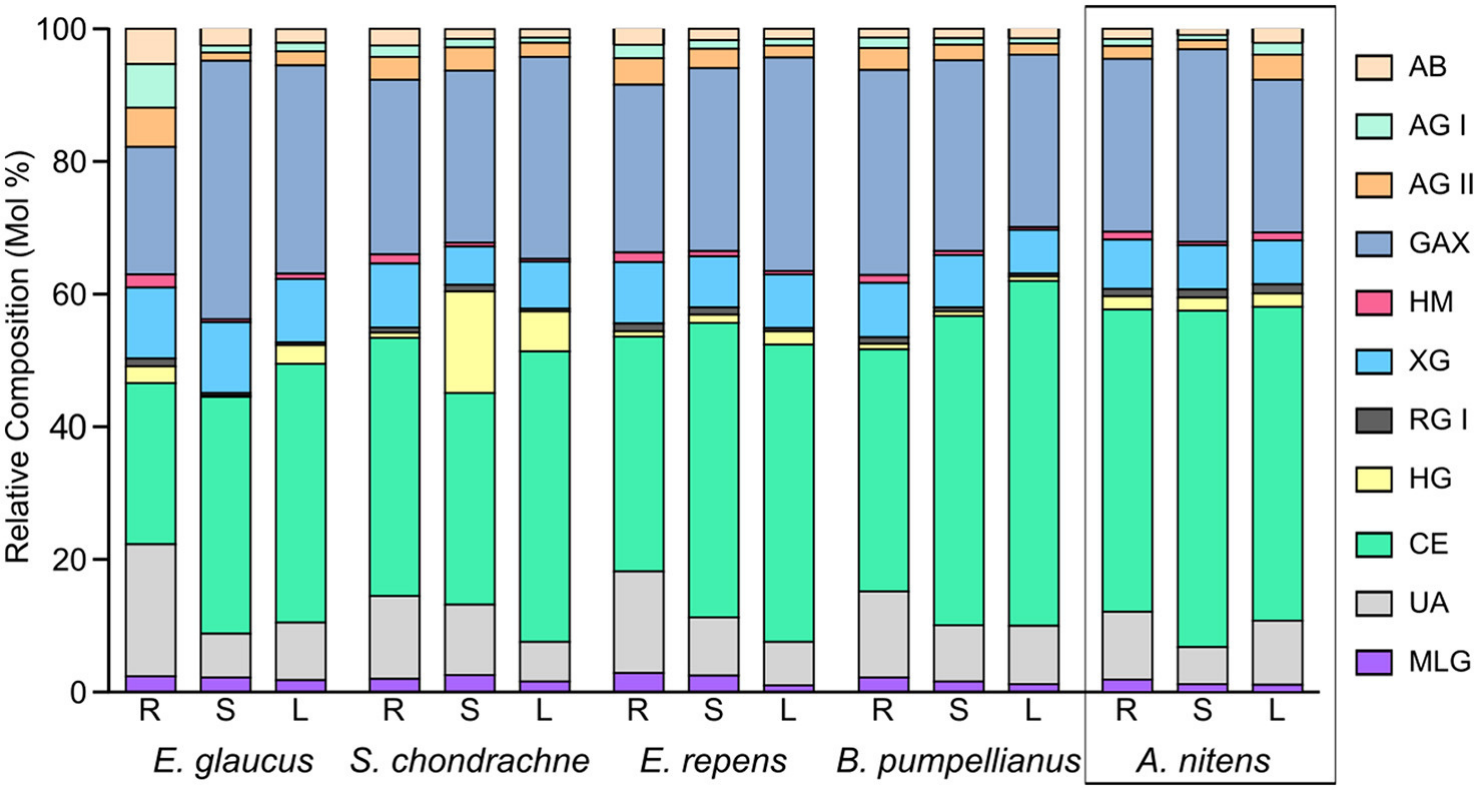
Averaged theorized polysaccharide composition for rhizome (R), stems (S), and leaves (L) from *A. nitens, E. glaucus, B. pumpellianus, S. chondrachne*, and *E. repens* (*n* = 2). Calculations were adapted from Pettolino et al. (2012). The following abbreviations were used for their respective polysaccharides: AB, arabinan; AG, arabinogalactan; GAX, glucuronoarabinoxylan; HM, heteromannan; XG, xyloglucan; RG, rhamnogalacturonan; HG, homogalacturonan; CE, cellulose; MLG, mixed linked glucan; UA, unassigned linkages.

The major polysaccharides estimated to be present in the cell walls of all the grass species (Figure 2) were cellulose (24–52%) and glucuronoarabinoxylans (19–39%). Xyloglucan was calculated to be present in significant amounts (6–11%), while minor levels of arabinans, arabinogalactans, heteromannans, mixed linked glucans, rhamnogalacturonans, and homogalacturonans were detected in the majority of the samples. The stems of *S. chondrachne* contained elevated levels of homogalacturonan (15%) whilst only comprising 1–3% of the cell walls in the tissues of other grass species. Homogeneity in the polysaccharide composition amongst the tissues in *A. nitens* and *E. repens* was observed, in which the relative composition of the polysaccharides were consistent between the tissues of the grass species.

### 3.5 Carbohydrate content of soils associated with greenhouse-grown Sweetgrass and other species collected from a nearby field

**Figure 3:**
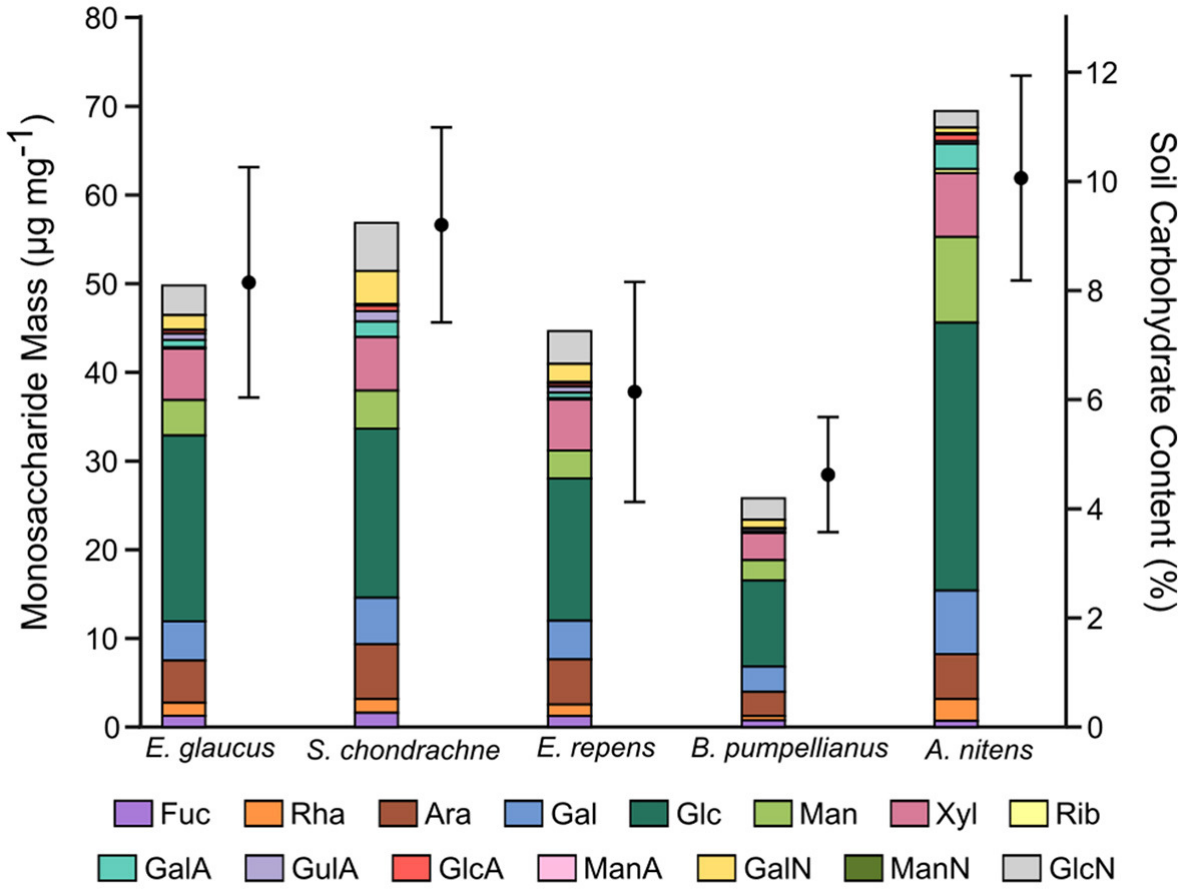
Carbohydrate content in greenhouse and field soil samples associated with Sweetgrass and other Poaceae grass species. Left *y*-axis: stacked bar chart of the masses for each monosaccharide present in the soil samples, determined by HPAEC-PAD analysis. Right *y*-axis: total carbohydrate content of each soil sample, determined by a colorimetric assay. Error bars represent the standard deviation of technical replicates (*n* = 2) from the colorimetric analysis of soil samples.

The total carbohydrate content was the highest amongst the greenhouse soil samples associated with the Sweetgrass plants at 10.1 ± 1.9% (Figure 3), whereby the field soil samples ranged from 4.6 ± 1.1 to 9.2 ± 1.8%. HPAEC-PAD analysis revealed that glucose was the most abundant monosaccharide within the soil samples, accounting for 30.2 μg mg^−1^ in the greenhouse samples and 9.7–20.9 μg mg^−1^ in the field samples (Figure 3). Further, significantly elevated amounts of glucose, galactose, mannose, xylose, and galacturonic acid were also seen in greenhouse soil samples (*P* < 0.05). In contrast, all field soil samples contained higher amounts of glucosamine in comparison to greenhouse soil, where this difference was significant (*P* < 0.05; Figure 3) in all except for the comparison between *B. pumpellianus* and greenhouse soils. Whereas, relatively consistent amounts of arabinose and xylose were seen amongst all soil samples, with lesser amounts of fucose, rhamnose, ribose, and other amino sugars and uronic acids.

### 3.6 16S microbial community analysis of greenhouse, field, and baseline soil samples

**Figure 4:**
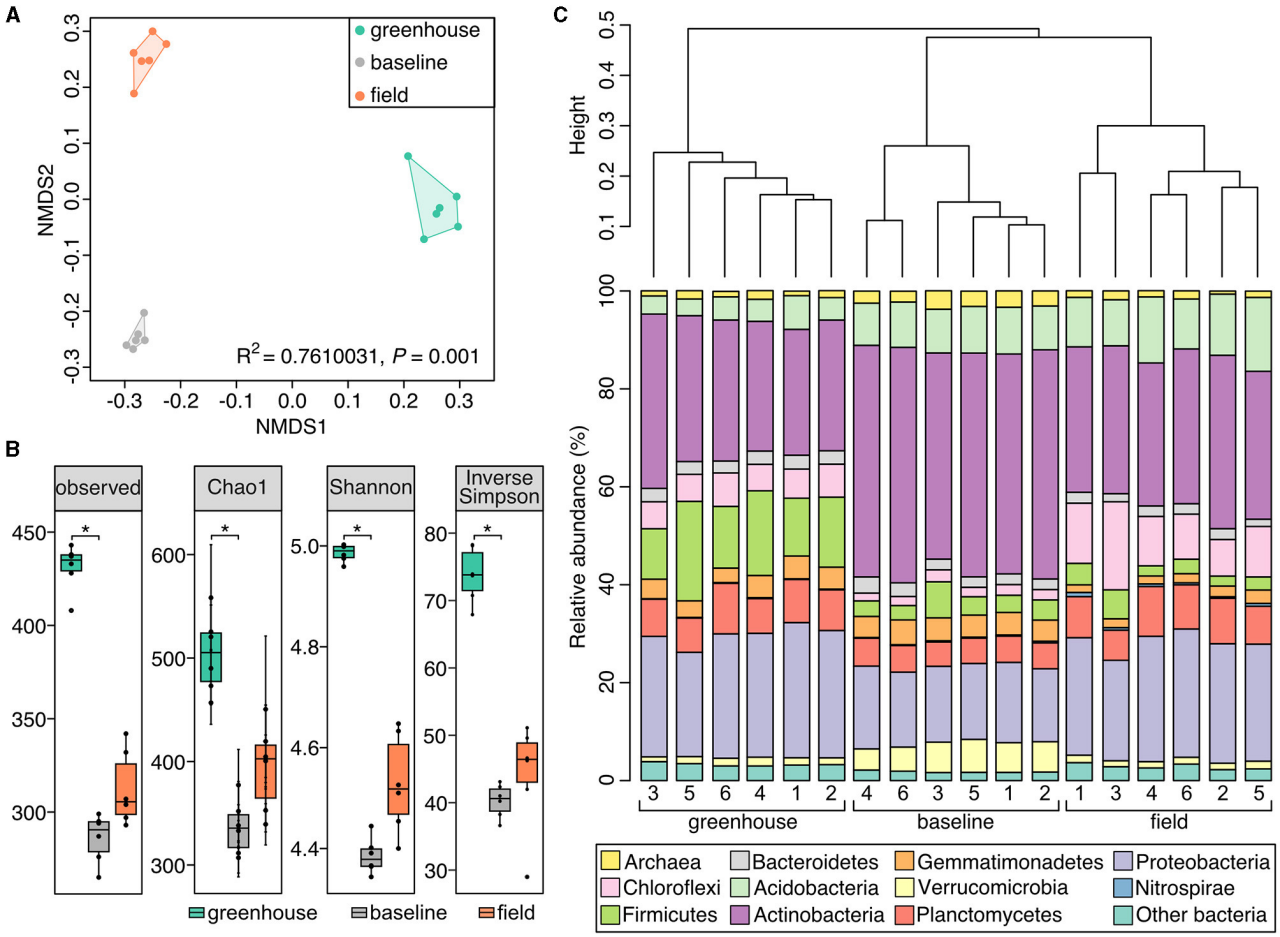
Soil microbial community composition differs between the three Piikani Nation sampling sites. **(A)** Non-metric multi-dimensional plot comparing the microbial communities of the soil samples collected from the greenhouse (teal), baseline (gray), and field (orange) sites. **(B)** Boxplots of the observed richness, Chao1, Shannon, and inversed Simpson diversity for each sample type. Boxplots and dots are colored to represent the different sample types: baseline (gray), greenhouse (teal), and field (orange). *Signifies a statistical difference (*P* < 0.05). **(C)** Dendrogram showing the similarity clustering of microbial community compositions between soil samples collected from greenhouse, baseline, and field plots in the Piikani Nation. Bar charts underneath, shows the relative community composition for each greenhouse, baseline, and field samples (*n* = 6) at the phylum level.

The greenhouse, field, and baseline soil samples significantly differed in their bacterial community composition (Figure 4A; PERMANOVA: *R*^2^ = 0.76, *P* = 0.001). Richness indices (observed, Chao1) were significantly higher in greenhouse soil samples (*P* < 0.05; Figure 4B) in comparison to baseline soil samples. Furthermore, soil bacterial alpha-diversity (Shannon and Simpson indices) at the genus level was also significantly higher in greenhouse soil (*P* < 0.05; Figure 4B) in comparison to baseline but not field soil samples. Although field soil samples did not show significant differences in bacterial diversity, these samples were marginally higher in richness and alpha-diversity values compared to baseline samples. Further, the dendrogram shown in Figure 4C illustrates the clustering pattern of the samples according to the three sampling sites, where field and baseline samples are most related to each other.

**Figure 5:**
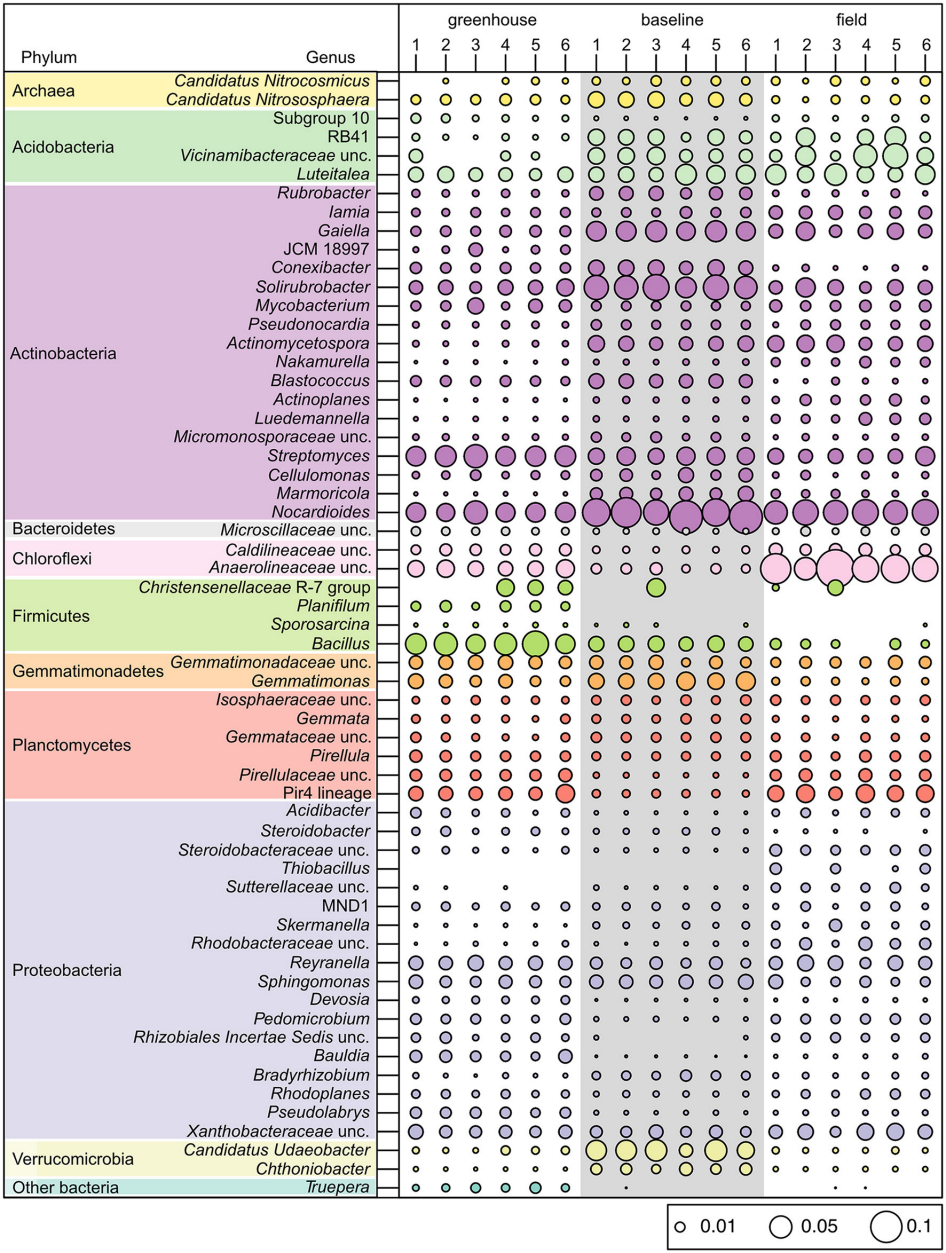
Bubble plot of different bacterial and archaeal genera that reached a minimum percentage of 1% relative read abundance in soil microbial communities collected from the greenhouse, baseline, and field sampling sites within the Piikani Nation (*n* = 6 for each sample type).

Amongst all soil samples analyzed, the most abundant bacterial phyla were Actinobacteria and Proteobacteria, where on average, these phyla comprised 42% of the greenhouse, 51% of the baseline, and 47% of the field soil microbial communities (Figure 4C). Many of the genera belonging to these phyla were seen to have similar relative abundances across the three sampling sites (e.g., *Iamia, Mycobacterium, Pseudonocardia, Reyranella, Sphingomonas*, and uncultured *Xanthobacteraceae*; Figure 5). The six greenhouse soil samples contained a higher relative abundance of Firmicutes in comparison to baseline and field soil samples (Figure 4C), as an increased proportion of *Bacillus* members was seen in the greenhouse soil and members of the *Planifilum* genera were only detected in this sample (Figure 5). Additionally, members belonging to the JCM 18977 (Actinobacteria), and *Truepera* genera (Deinococcota) were also only detected in greenhouse soil samples. In contrast, there was a higher relative abundance of Chloroflexi seen in the field soil samples (Figure 4C), where uncultured *Anaerolineaceae* were drastically increased within these samples (Figure 5). The baseline field samples contained greater relative abundances of Verrucomicrobia and Actinobacteria, in comparison to the greenhouse and field soil samples (Figure 4C). Members belonging to the candidatus *Udaeobacter* and *Chtoniobacter* genera, and *Gaiella, Conexibacter, Solirubrobacter*, and *Nocardioides* genera were increased (Figure 5), an observation largely attributed to the increased relative abundances of Verrucomicrobia and Actinobacteria, respectively.

### 3.7 Soil chemistry

Soil chemical analysis revealed significantly higher total nitrogen, total carbon, total organic carbon, and NH_3_-N in the greenhouse compared to baseline soils. Greenhouse soils had significantly higher NO_3_-N than both baseline and field soils. Field soils had significantly higher pH values compared to the greenhouse soils (Table 3).

**Table 3:** Soil physiochemical parameters of baseline, field, and greenhouse soils affected by Sweetgrass (*A. nitens*).

**Soil type**	**TN (g kg^−1^)**	**TC (g kg^−1^)**	**TOC (g kg^−1^)**	**pH**	**NO_3_-N (μg g^−1^)**	**NH_3_(μg g^−1^)**
Greenhouse	0.071 ± 0.0025 (a)	1.67 ± 0.0879 (a)	1.57 ± 0.07 (a)	7.22 ± 0.07 (a)	482.12 ± 44.94 (a)	22.46 ± 4.13 (a)
Baseline	0.024 ± 0.0003 (b)	0.39 ± 0.0036 (b)	0.32 ± 0.01 (b)	8.15 ± 0.01 (ab)	3.34 ± 0.70 (b)	3.29 ± 0.11 (b)
Field	0.047 ± 0.0025 (ab)	0.63 ± 0.0274 (ab)	0.54 ± 0.03 (b)	8.47 ± 0.03 (b)	3.08 ± 0.25 (b)	7.68 ± 0.53 (ab)

Parameters include TN, total nitrogen; TC, total carbon; TOC, total organic carbon; pH, NO3-N, nitrate; NH3-N, ammonia (*n* = 6). Different letters (a, b) signifies a statistical difference (*P* < 0.05). *n* = 6.

## 4 Discussion

Sweetgrass (*A. nitens*) and the four different field grass species analyzed within this study, are all Graminacous monocots belonging to the Poaceae family. Therefore, it is not surprising to see that these grass species have a similar polysaccharide profile to each other and to other Poaceae members (Smith and Harris, 1999; Gibeaut et al., 2005). In the glycomics analysis conducted here, cellulose and GAX were determined to be the predominant polysaccharides in Sweetgrass and the field grass species, where each species contained smaller proportions of other hemicelluloses (i.e., mixed-linkage glucans, xyloglucans, mannans) and pectic polysaccharides (i.e., homogalacturonan, rhamnogalacturonan) (Figure 2). In comparison, the cellulose content of Sweetgrass stems was previously reported to be 70.4% through gravimetric analysis (Dalmis et al., 2020). Xylan polysaccharides, such as heteroxylans, arabinoxylans, and GAX, typically represent 20–30% of the total cell wall content within Poaceae grass species (Hatfield et al., 2017), and were determined here to represent 19–32% of the tissue cell walls of various Poaceae species. Within the primary cell walls, Poaceae GAX typically contains more Ara*f* residues within the GAX backbone (Scheller and Ulvskov, 2010), where secondary cell walls also contain some GlcA*p* or MeGlcA*p* substituents as well (Peña et al., 2016). These Ara*f* residues are often esterified with coumaric or ferulic acids (Buanafina, 2009). In comparison, woody and herbaceous eudicots contain glucuronoxylans (Peña et al., 2016). It is hypothesized that commelinids, including Poaceae grasses, incorporate Ara*f* residues into glucuronoxylan polysaccharides to form GAX, whereby this addition may provide GAX with certain physical properties, allowing it to replace the function of xyloglucan and/or pectin as a cross linker (Carpita, 1996). The walls of the Poaceae are further distinguished by the presence of variable amounts of (1,3; 1,4)-linked β-glucans (Carpita and Gibeaut, 1993; Smith and Harris, 1999), where these are widely distributed as non-cellulosic matrix phase polysaccharides within the cell walls (Burton and Fincher, 2009). Here, the presence of the 3-Glc*p* and 4-Glc*p* linkages throughout all Sweetgrass and field grass tissue sections, indicates the presence of (1,3; 1,4)-glucans (Supplementary Figure S1; Supplementary Tables S3, S4).

The only striking difference in carbohydrate composition was seen in the stems of *S. chondrachne*, as these samples contained elevated levels of GalA and GlcA uronic acids (Figures 1A, 2; Table 2). This suggested an increased amount of HG within the stems of this plant. Pectins make up ~5% of growing cell walls in Poaceae species (Vogel, 2008), whereas it constitutes ~0.1% in mature cell walls (Ishii, 1997). Potentially, this increased abundance of pectin seen within *S. chondrachne* signifies that this plant was actively growing, as pectins allow for primary cell wall extension and plant growth to occur (Wolf and Greiner, 2012).

Carbohydrates commonly account for 5–20% of soil organic matter (Swincer et al., 1969). Here, the carbohydrate content of the soil AIR samples ranged from 4.6 to 10.1% (Figure 2), where the highest value was seen in the greenhouse soil. Greenhouse soil samples were enriched with statistically higher amounts of mannose, galactose, and galacturonic acid (Figure 3). The addition of bloodmeal has been previously shown to increase the total organic carbon within the soil (Ciavatta et al., 1997), positively impacting the relationship between soil microbial diversity and biomass (Bastida et al., 2021). Therefore, as bloodmeal was used to help initiate the greenhouse nursery bed soil, it is most likely that the fertilizer increased available nitrogen and organic carbon in the greenhouse soil (Table 3).

The available nutrients of each soil supported a particular microbial community that utilized enzymes to deconstruct available organic carbon (i.e., polysaccharides) into usable energy (i.e., monosaccharides). The high amount of glucose present with each sample is indicative of cellulose and hemicellulose deposition. Further, the presence of mannose, xylose, galactose, and arabinose within these soil samples suggests a role in hemicellulose turnover, as all four monosaccharides are present in hemicellulose polysaccharides. Proteobacteria, Firmicutes, Acidobacteria, Bacteroidetes, Actinobacteria, and Verrucomicrobia are all known phyla that contain cellulolytic members (López-Mondéjar et al., 2019) and were represented in each of the soils. The higher amount of monosaccharides related to hemicellulose turnover in the greenhouse soil again suggests a higher rate of turnover, which is supported by an increased abundance of members shown to deconstruct hemicelluloses, such as *Bacillus* and *Streptomyces*. Assuming that the litter decomposition accounts for a fraction of soil monosaccharides, the abundances of GalA and GlcA observed can also be attributed to pectin degradation (Caffall and Mohnen, 2009) most likely by Proteobacteria, Firmicutes, and Bacteroidetes members. Whereas some of the GlcA may also come from bacterial cell wall polysaccharides (Vinnitskiy et al., 2015). As the amount of GlcA and GalA were higher in the greenhouse samples, and variable in the field samples, it suggests that there is higher cell and litter deposition and turnover in the greenhouse soil samples. In contrast, very little ManA and GulA were detected in the soil samples, where GulA was higher in the field samples. GulA and ManA are commonly used as indicators for the presence of alginate, a common polysaccharide in bacterial biofilms (Gheorghita et al., 2022). The production of extracellular polysaccharides by bacterial species, can facilitate prolonged cellular hydration and nutrient resupply in dry soil environments (Or et al., 2007). Additionally, this can affect the soil structure by promoting water retention and aggregate formation (Chenu and Roberson, 1996; Henao and Mazeau, 2009). Lastly, amino sugars are present in soil and account for a generous fraction of soil nitrogen content (Indorf et al., 2011). Amino sugars (i.e., MurN, GlcN, GalN, ManN) are also present in the cell wall components of both bacteria and fungi in the form of peptidoglycan and chitin, respectively (Joergensen, 2018). Here, GalN and GlcN were detected in each sample with higher amounts of both being seen in field soil samples (Figure 3), which can be presumably traced to bacterial and fungal cell wall metabolism (Amelung et al., 1999).

Correlating the soil microbiome with the plant and soil glycomes is challenging as the interactions are highly complex and variables in the current study were limited. The composition of soil microbiomes can vary to the extent where there is not a “typical” soil microbiome that can be found amongst different soil types (e.g., clay, loam). This statement is also true if multiple soil samples are collected from sites that are centimeters apart, due to the spatial variability of soil and specific characteristics of each sampling site (O'Brien et al., 2016). Thus, inconsistency seen between the total carbohydrate content of the field soil samples may be partially explained by differences in the soil sampling sites, where soil samples collected from the field site were inconsistent in the distances between sampling locations. In contrast, all six soil samples collected from the greenhouse were gathered from the same 1 m^2^ nursery bed. Furthermore, the composition of above-ground plant species cannot be used to predict the soil microbiome composition, as many soil bacteria may be associated with a broad range of plant taxa. However, taxonomic analysis of soil samples can provide insight regarding ecological services that may be occurring in the soil (Banerjee and Van Der Heijden, 2023). These insights could potentially be used as biological indicators to assess soil conditions and health.

Several other factors such as; soil pH, nitrogen availability, soil organic matter, moisture, season, and temperature, can directly or indirectly influence the spatial structure of soil microbial communities, with pH having a profound impact on bacteria (Rousk et al., 2010; Murphy et al., 2016). A less alkaline pH in the greenhouse soil (Table 3) could contribute to the differences in community structure between the greenhouse, field and baseline soils (Figures 4, 5). While soil moisture was not measured in the field, the plants were collected during a drought (Supplementary Table S6) and soils were assessed to contain 0% moisture in the lab. In contrast, greenhouse soil samples were watered to field moist conditions. Previous studies have shown that drier soils result in more restrained microbial dispersion, as microbes are limited to soil pores (Jansson and Hofmockel, 2018). In terms of soil microbial members, the relative abundance of Actinobacteria has been shown to increase under dry conditions, where this phylum becomes established as a dominant group in the soil environment (Naylor et al., 2017; Preece et al., 2019). In contrast, the abundance of Proteobacteria is negatively correlated with drought conditions (Preece et al., 2019). Here, an elevated abundance of Actinobacteria and a decreased abundance of Proteobacteria were seen in baseline soil samples (Figure 4C). Further, the relative abundances of these two phyla were also slightly higher in field soil samples, in comparison to greenhouse samples.

Soil amendments, such as the use of organic fertilizers (i.e., bloodmeal) have been previously shown to increase the relative abundances of Firmicutes and Proteobacteria within a soil microbial community (Schlatter et al., 2019). Here, the abundance of Proteobacteria was similar between the greenhouse and field soils, and was significantly reduced in the baseline soils. However, the relative abundance of Firmicutes increased in greenhouse soil samples (Figure 4C), which is likely the result of the bloodmeal amendments (Francioli et al., 2016). Specifically, there was a drastic increase in the abundance of Firmicutes members belonging to the *Bacillus* genus (Figure 5). This result may reflect a “healthy soil microbiome” as *Bacillus* species are commonly associated with beneficial effects within a soil ecosystem, such as phosphate solubilization and siderophore production to aid in iron uptake (Fierer and Jackson, 2006). Additionally, *Bacillus* members are also associated with the promotion of plant growth (Lugtenberg and Kamilova, 2009) and disease suppression (Zhou et al., 2023), having a direct effect on plant health.

## 5 Conclusion

Through a collaboration with a climate-focused charity and a First Nations community, glycomics traits associated with greenhouse-grown Sweetgrass were determined to predominantly contain cellulose and GAX polysaccharides, with an even monosaccharide distribution between the rhizomes, stems, and leaves. Within the soil microbial communities associated with Sweetgrass, an increased relative abundance of Firmicutes members was seen in comparison to the baseline and field soils and bacterial members belonging to the *Planifilum, Truepera*, and JCM 18977 were only detected in the greenhouse soil samples. These observations, along with the traditional knowledge of Piikani Nation provided by knowledge holders, will help inform future work regarding the re-introduction of greenhouse-grown Sweetgrass back into the environment. Further studies will be required to see if the traits associated with the greenhouse cultivated Sweetgrass persist in plants grown and harvested from the field.

## Data availability statement

The data presented in the study are deposited in the NCBI repository, BioProject Accession No: PRJNA1110276.

## Author contributions

MK: Writing – original draft, Visualization, Methodology, Data curation. BB: Writing – review & editing, Methodology. NH: Writing – review & editing, Methodology. XX: Writing – review & editing, Methodology. KL: Writing – review & editing, Methodology. PN: Writing – review & editing, Methodology. EH: Writing – review & editing, Methodology. MV: Writing – review & editing, Methodology. BW: Writing – review & editing, Resources, Methodology, Conceptualization. LK: Writing – review & editing, Visualization, Methodology. NP: Writing – review & editing, Resources, Funding acquisition. WB: Writing – review & editing, Resources, Funding acquisition. LL: Writing – review & editing, Funding acquisition. TM: Writing – review & editing, Supervision, Methodology, Funding acquisition. PT: Writing – review & editing, Funding acquisition. MG: Writing – review & editing, Supervision, Funding acquisition. DA: Writing – review & editing, Supervision, Funding acquisition, Conceptualization.
