# Supplementary material for: Comparative analysis of the soil microbiome and carbohydrate content of Anthoxanthum nitens (Sweetgrass) and other Poaceae grass tissues and associated soils
Source: Front Microbiol. 2025 Apr 28;15:1384204. doi: 10.3389/fmicb.2024.1384204 (PMC12067597; doi:10.3389/fmicb.2024.1384204)
Supplement: Supplementary file 1 [file Data_Sheet_1.pdf]

Supplementary Material

Comparative analysis of the soil microbiome and carbohydrate content of *Anthoxanthum nitens* (Sweetgrass) tissues and associated soils

Marissa L. King, Barinder Bajwa, Naomi Hanna, Xiaohui Xing, Kristin E. Low, Patrick Neuberger, Erin Hall, Michael Veltri, Brett Weighill, Leeann Klassen, Noreen Plain Eagle, William Big Bull, Laura S. Lynes, Tony Montina, Philippe J. Thomas, Monika A. Gorzelak, D. Wade Abbott*

*** Correspondence:** D. Wade Abbott: wade.abbott@agr.gc.ca

1 Supplementary Figures and Tables

Table S1 Gradient conditions used for the HPLC separation of neutral and uronic acids.

Time (min)	100 mM NaOH	100 mM NaOH 1 M NaOAc	H2O
	A (%)	B (%)	C (%)
0	20	0	80
20	19	1	80
20.1	15	5	0
60	80	20	0
60.1	100	0	0
70	100	0	0
70.1	20	0	80
80	20	0	80

Table S2 Gradient conditions used for the HPLC separation of amino sugars.

Time (min)	100 mM NaOH	100 mM NaOH 1 M NaOAc	H2O
	A (%)	B (%)	C (%)
0	10	0	90
20	10	0	90
20.1	100	0	0
30	100	0	0
30.1	10	0	90
40	10	0	90

Table S3 Relative abundances (Mol%) of glycosidic linkages identified from the rhizome, stems, and leaves from the cell walls of *A. nitens* (n=2) via GC-MS. Tr. = Trace (Mol % < 0.5%)

Tissue	Rhizome						Stems						Leaves					
Bio Rep	1	2	3	4	5	6	1	2	3	4	5	6	1	2	3	4	5	6
t-Ara*p*	Tr.	Tr.	Tr.	Tr.	Tr.	Tr.	Tr.	Tr.	Tr.	Tr.	Tr.	Tr.	Tr.	Tr.	Tr.	Tr.	Tr.	Tr.
t-Ara*f*	3.5	3.8	2.8	3.5	3.9	2.9	3.5	3.2	4.5	3.1	3.4	3.2	4.5	5	3.5	3.4	4.2	4.8
2-Ara*f*	0.6	0.5	0.5	Tr.	0.8	Tr.	Tr.	Tr.	Tr.	Tr.	0.6	0.5	0.7	0.6	Tr.	0.6	0.6	0.6
3-Ara*f*	0.9	0.9	0.7	0.7	1.1	0.7	0.7	0.7	0.7	0.6	0.7	0.6	0.9	0.9	0.6	0.6	0.6	0.7
5-Ara*f*	1	0.9	0.6	0.5	1.3	0.6	0.6	0.7	0.6	0.6	0.5	0.6	2	1.9	0.9	0.8	0.9	1
2,3,5-Ara*f*	0.6	Tr.	Tr.	Tr.	Tr.	0.5	Tr.	Tr.	Tr.	Tr.	Tr.	Tr.	Tr.	Tr.	Tr.	Tr.	Tr.	Tr.
t-Fuc*p*	Tr.	Tr.	Tr.	Tr.	Tr.	Tr.	Tr.	Tr.	Tr.	Tr.	Tr.	Tr.	Tr.	Tr.	Tr.	Tr.	Tr.	Tr.
t-Galp	1.6	1.5	1.1	1.2	2.4	1.3	1.1	1	1.1	0.9	1.1	1	1.5	1.4	0.9	0.9	1	1.2
2-Galp	Tr.	Tr.	Tr.	Tr.	Tr.	Tr.	Tr.	Tr.	Tr.	Tr.	Tr.	Tr.	Tr.	Tr.	Tr.	Tr.	Tr.	Tr.
3-Galp	0.6	0.5	Tr.	Tr.	0.9	Tr.	0.5	0.5	0.5	Tr.	Tr.	0.6	0.9	0.9	0.5	0.5	Tr.	0.5
4-Gal*p*	Tr.	Tr.	Tr.	Tr.	Tr.	Tr.	Tr.	Tr.	Tr.	Tr.	Tr.	Tr.	Tr.	Tr.	Tr.	Tr.	Tr.	Tr.
6-Gal*p*	Tr.	Tr.	Tr.	Tr.	Tr.	Tr.	Tr.	Tr.	Tr.	Tr.	Tr.	Tr.	Tr.	Tr.	Tr.	Tr.	Tr.	Tr.
2,6-Gal*p*	Tr.	Tr.	Tr.	Tr.	Tr.	Tr.	Tr.	Tr.	Tr.	Tr.	Tr.	Tr.	Tr.	Tr.	Tr.	Tr.	Tr.	Tr.
3,4-Gal*p*	0.5	Tr.	Tr.	Tr.	0.5	Tr.	Tr.	Tr.	Tr.	Tr.	Tr.	Tr.	0.5	0.6	0.5	0.5	Tr.	Tr.
3,6-Gal*p*	Tr.	Tr.	Tr.	Tr.	0.8	Tr.	Tr.	Tr.	Tr.	Tr.	Tr.	Tr.	1.1	0.7	Tr.	Tr.	Tr.	Tr.
4,6-Gal*p*	Tr.	Tr.	Tr.	Tr.	Tr.	Tr.	Tr.	Tr.	Tr.	Tr.	Tr.	Tr.	Tr.	Tr.	Tr.	Tr.	Tr.	Tr.
2,3,6-Gal*p*	Tr.	Tr.	Tr.	Tr.	Tr.	Tr.	Tr.	Tr.	Tr.	Tr.	Tr.	Tr.	Tr.	Tr.	Tr.	Tr.	Tr.	Tr.
2,4,6-Gal*p*	Tr.	Tr.	Tr.	Tr.	Tr.	Tr.	Tr.	Tr.	Tr.	Tr.	Tr.	Tr.	Tr.	Tr.	Tr.	Tr.	Tr.	Tr.
3,4,6-Gal*p*	Tr.	Tr.	Tr.	Tr.	Tr.	Tr.	Tr.	Tr.	Tr.	Tr.	Tr.	Tr.	0.5	Tr.	Tr.	Tr.	Tr.	Tr.
2,3,4,6-Gal*p*	Tr.	Tr.	Tr.	Tr.	Tr.	Tr.	Tr.	Tr.	Tr.	Tr.	Tr.	Tr.	Tr.	Tr.	Tr.	Tr.	Tr.	Tr.
2,4-Glc*p*+ 2,4-Gal*p*	0.5	0.5	Tr.	Tr.	Tr.	Tr.	Tr.	Tr.	Tr.	Tr.	Tr.	Tr.	Tr.	0.5	0.5	Tr.	Tr.	Tr.
t-Glc*p*	2.3	2	1.8	1.5	3.1	1.7	1.2	0.9	1	0.9	1.1	0.8	2.1	2.2	1.8	1.6	1.5	2.3
2-Glc*p*	Tr.	Tr.	Tr.	Tr.	1	Tr.	Tr.	Tr.	Tr.	Tr.	Tr.	Tr.	Tr.	0.5	0.5	Tr.	Tr.	Tr.
3-Glc*p*	0.9	1.2	0.7	0.7	1.4	0.9	0.7	0.5	0.7	0.6	0.6	Tr.	0.6	0.5	Tr.	Tr.	Tr.	Tr.

4-Glc*p*	49	51.2	50.3	51.4	42.8	52.5	53.6	54.3	53.3	52.1	55.1	53.1	50.1	51.3	53.1	53.4	48.6	55.6
6-Glc*p*	Tr.	Tr.	Tr.	Tr.	0.5	Tr.	Tr.	Tr.	Tr.	Tr.	Tr.	Tr.	Tr.	0.5	0.5	Tr.	Tr.	Tr.
2,3-Glc*p*	Tr.	Tr.	Tr.	Tr.	Tr.	Tr.	Tr.	Tr.	Tr.	Tr.	Tr.	Tr.	Tr.	Tr.	Tr.	Tr.	Tr.	Tr.
3,4-Glc*p*	0.7	0.7	0.6	0.5	0.5	0.6	0.5	0.5	0.5	0.5	0.5	0.6	0.6	0.9	0.8	0.7	0.5	0.7
3,6-Glc*p*	Tr.	Tr.	Tr.	Tr.	Tr.	Tr.	Tr.	Tr.	Tr.	Tr.	Tr.	Tr.	Tr.	Tr.	Tr.	Tr.	Tr.	Tr.
4,6-Glc*p*	3.1	2.6	2.5	1.5	2.8	2.6	2.1	2	2.1	2.2	1.7	2.5	2.1	2.6	3	2.8	2.2	1.9
2,3,6-Glc*p*	Tr.	Tr.	Tr.	Tr.	Tr.	Tr.	Tr.	Tr.	Tr.	Tr.	Tr.	Tr.	Tr.	Tr.	Tr.	Tr.	Tr.	Tr.
2,4,6-Glc*p*	Tr.	Tr.	Tr.	Tr.	Tr.	Tr.	Tr.	Tr.	Tr.	Tr.	Tr.	Tr.	Tr.	Tr.	Tr.	Tr.	Tr.	Tr.
3,4,6-Glc*p*	Tr.	Tr.	Tr.	Tr.	Tr.	Tr.	Tr.	Tr.	Tr.	Tr.	Tr.	Tr.	Tr.	Tr.	Tr.	Tr.	Tr.	Tr.
2,3,4,6-Glc*p*	1.1	0.9	0.7	Tr.	0.5	0.9	Tr.	Tr.	Tr.	Tr.	Tr.	0.5	Tr.	0.7	0.7	0.5	Tr.	Tr.
t-Man*p*	0.7	0.6	Tr.	Tr.	1	Tr.	Tr.	Tr.	Tr.	Tr.	Tr.	Tr.	Tr.	0.6	Tr.	Tr.	Tr.	Tr.
3-Man*p*	Tr.	Tr.	Tr.	Tr.	Tr.	Tr.	Tr.	Tr.	Tr.	Tr.	Tr.	Tr.	Tr.	Tr.	Tr.	Tr.	Tr.	Tr.
4-Man*p*	0.6	Tr.	0.5	Tr.	0.7	Tr.	Tr.	Tr.	Tr.	Tr.	Tr.	Tr.	0.5	0.5	Tr.	Tr.	Tr.	Tr.
6-Man*p*	Tr.	Tr.	Tr.	Tr.	Tr.	Tr.	Tr.	Tr.	Tr.	Tr.	Tr.	Tr.	Tr.	Tr.	Tr.	Tr.	Tr.	Tr.
2,4-Man*p*	Tr.	Tr.	Tr.	Tr.	Tr.	Tr.	Tr.	Tr.	Tr.	Tr.	Tr.	Tr.	Tr.	Tr.	Tr.	Tr.	Tr.	Tr.
2,6,Man*p*	0.5	Tr.	Tr.	Tr.	1.1	Tr.	Tr.	Tr.	Tr.	Tr.	Tr.	Tr.	Tr.	Tr.	Tr.	Tr.	Tr.	Tr.
3,4-Man*p*	Tr.	Tr.	Tr.	Tr.	Tr.	Tr.	Tr.	Tr.	Tr.	Tr.	Tr.	Tr.	Tr.	Tr.	Tr.	Tr.	Tr.	Tr.
3,6-Man*p*	Tr.	Tr.	Tr.	Tr.	Tr.	Tr.	Tr.	Tr.	Tr.	Tr.	Tr.	Tr.	Tr.	Tr.	Tr.	Tr.	Tr.	Tr.
4,6-Man*p*	Tr.	Tr.	Tr.	Tr.	Tr.	Tr.	Tr.	Tr.	Tr.	Tr.	Tr.	Tr.	Tr.	Tr.	Tr.	Tr.	Tr.	Tr.
2,3,6-Man*p*	Tr.	Tr.	Tr.	Tr.	Tr.	Tr.	Tr.	Tr.	Tr.	Tr.	Tr.	Tr.	Tr.	Tr.	Tr.	Tr.	Tr.	Tr.
2,4,6-Man*p*	Tr.	Tr.	Tr.	Tr.	Tr.	Tr.	Tr.	Tr.	Tr.	Tr.	Tr.	Tr.	Tr.	Tr.	Tr.	Tr.	Tr.	Tr.
3,4,6-Man*p*	Tr.	Tr.	Tr.	Tr.	Tr.	Tr.	Tr.	Tr.	Tr.	Tr.	Tr.	Tr.	Tr.	Tr.	Tr.	Tr.	Tr.	Tr.
2,3,4,6-Man*p*	Tr.	Tr.	Tr.	Tr.	Tr.	Tr.	Tr.	Tr.	Tr.	Tr.	Tr.	Tr.	Tr.	Tr.	Tr.	Tr.	Tr.	Tr.
t-Rha*p*	Tr.	Tr.	Tr.	Tr.	Tr.	Tr.	Tr.	Tr.	Tr.	Tr.	Tr.	Tr.	Tr.	Tr.	Tr.	Tr.	Tr.	Tr.
2-Rha*f*	Tr.	Tr.	Tr.	0.5	Tr.	Tr.	0.6	0.6	0.6	Tr.	Tr.	Tr.	Tr.	Tr.	Tr.	Tr.	Tr.	Tr.
4-Rha*p*	Tr.	Tr.	Tr.	Tr.	Tr.	Tr.	Tr.	Tr.	Tr.	Tr.	Tr.	Tr.	Tr.	Tr.	Tr.	Tr.	Tr.	Tr.
2,3-Rha*p*	Tr.	Tr.	Tr.	Tr.	Tr.	Tr.	Tr.	Tr.	Tr.	Tr.	Tr.	Tr.	Tr.	Tr.	Tr.	Tr.	Tr.	Tr.
2,4-Rha*p*	Tr.	0.5	Tr.	Tr.	Tr.	Tr.	Tr.	Tr.	Tr.	Tr.	Tr.	Tr.	0.7	0.5	Tr.	Tr.	Tr.	Tr.
3,4-Rha*p*	Tr.	Tr.	Tr.	Tr.	Tr.	Tr.	Tr.	Tr.	Tr.	Tr.	Tr.	Tr.	Tr.	Tr.	Tr.	Tr.	Tr.	Tr.
2,3,4-Rha*p*	Tr.	Tr.	Tr.	Tr.	0.5	Tr.	Tr.	Tr.	Tr.	Tr.	Tr.	Tr.	Tr.	Tr.	Tr.	Tr.	Tr.	Tr.
t-Xyl*p*	0.7	0.7	0.8	0.8	0.9	0.7	0.9	0.8	0.8	0.7	0.8	0.7	0.6	0.8	0.7	0.9	0.8	0.8

2-Xyl*p*	1.3	1.6	1.8	1.8	1.5	1.7	1.7	1.8	1.5	1.7	1.7	1.7	1.2	1.1	1.3	1.5	1.4	1.3
4-Xyl*p*	11.4	13.4	16.5	15.7	10.1	15.6	14.5	16.5	13.9	15.5	14.8	15.9	10.1	9.3	12.4	14.4	13.2	11.3
2,4-Xyl*p*	1.3	1.3	1.4	1.3	1.4	1.2	1.8	1.7	1.4	1.7	1.6	1.8	1.4	1.4	1.5	1.7	1.5	1.2
3,4-Xyl*p*	5.8	5.5	5.1	5.2	5.6	4.9	6.1	6.6	6.3	6.3	6.2	6.6	6.3	5.8	6.1	6.1	5.9	5.7
2,3,4-Xyl*p*	1.6	0.8	0.8	Tr.	0.7	1.2	0.9	0.9	0.9	1.7	0.5	1.9	0.6	0.6	1.8	1.6	1	Tr.
t-GalA*p*	Tr.	Tr.	0.5	0.8	0.6	Tr.	0.5	Tr.	Tr.	0.7	0.6	Tr.	0.6	0.5	Tr.	Tr.	1.4	0.5
2-GalA*p*	Tr.	Tr.	Tr.	Tr.	Tr.	Tr.	Tr.	Tr.	Tr.	Tr.	Tr.	Tr.	Tr.	Tr.	Tr.	Tr.	Tr.	Tr.
3-GalA*p*	Tr.	Tr.	Tr.	Tr.	Tr.	Tr.	Tr.	Tr.	Tr.	Tr.	Tr.	Tr.	Tr.	Tr.	Tr.	Tr.	Tr.	Tr.
4-GalA*p*	1.2	0.9	1.4	3.7	2.1	0.5	1.8	1	2.5	3.3	2.8	0.9	2.4	1.6	1.7	0.9	5.5	1.7
3,4-GalA*p*	Tr.	Tr.	Tr.	0.6	0.6	Tr.	Tr.	Tr.	Tr.	Tr.	Tr.	Tr.	Tr.	Tr.	Tr.	Tr.	Tr.	Tr.
2,4-GlcA*p*+ 2,4-GalA*p*	Tr.	Tr.	Tr.	Tr.	Tr.	Tr.	Tr.	Tr.	Tr.	Tr.	Tr.	Tr.	Tr.	Tr.	Tr.	Tr.	Tr.	Tr.
t-GlcA*p*	1.8	1.8	2.3	4.1	2.6	1.7	2	1.4	2.2	2	2.1	1.4	2.3	2	2	1.4	3.4	2.1
2-GlcA*p*	Tr.	Tr.	Tr.	Tr.	Tr.	Tr.	Tr.	Tr.	Tr.	Tr.	Tr.	Tr.	Tr.	Tr.	Tr.	Tr.	Tr.	Tr.
3-GlcA*p*	Tr.	Tr.	Tr.	Tr.	Tr.	Tr.	Tr.	Tr.	Tr.	Tr.	Tr.	Tr.	Tr.	Tr.	Tr.	Tr.	Tr.	Tr.

Note: Two separate experiments were conducted for each sample.

Formatted: English (Canada)

Table S4 Relative abundances (Mol%) of glycosidic linkages identified from the rhizome (R), stems (S), and leaves (L) from the cell walls of *E. glaucus*, *B. pumpellianus*, *S. chondrachne*, and *E. repens* via GC-MS. Tr. = Trace (Mol % < 0.5%)

Name	*E. glaucus*			*B. pumpellianus*			*S. chondrachne*			*E. repens*								
Tissue	R	S	L	R	S	L	R	S	L	R			S			L		
Bio Rep	1	1	1	1	1	1	1	1	1	1	2	3	1	2	3	1	2	3
t-Ara*p*	Tr.	Tr.	Tr.	0.5	Tr.	Tr.	0.7	Tr.	Tr.	0.5	0.5	Tr.	Tr.	Tr.	Tr.	Tr.	Tr.	Tr.
t-Ara*f*	5.6	3.1	3.1	4.9	3.5	3.3	5.1	3.5	3.6	5	3.4	3.5	3.6	4.2	2.8	2.6	3.4	3.5
2-Ara*f*	0.9	0.5	0.8	1.1	0.8	0.6	1	0.9	1	1.3	1.2	1.1	1.1	0.8	0.7	0.8	0.8	0.8
3-Ara*f*	1.7	0.7	1.2	1.6	1.1	1.1	1.5	1.4	0.7	1.8	1.6	1.6	1.6	1.5	1.1	0.9	0.9	0.9
5-Ara*f*	1.3	0.5	0.9	0.7	0.6	0.6	0.9	1.1	0.9	1.2	1.1	0.7	0.7	1.3	0.6	0.6	1	0.8
2,3,5-Ara*f*	2	1.5	0.6	Tr.	Tr.	Tr.	0.8	Tr.	Tr.	1	0.8	Tr.	Tr.	0.6	Tr.	Tr.	0.7	Tr.

t-Fuc*p*	Tr.	Tr.	Tr.	Tr.	Tr.	Tr.	Tr.	Tr.	Tr.	Tr.	Tr.	Tr.	Tr.	Tr.	Tr.	Tr.	Tr.	Tr.
t-Gal*p*	2.5	0.6	1.2	1.9	1.3	1.1	1.6	1.9	1.3	2.1	2	1.3	1.3	1.4	0.9	0.8	0.8	0.9
2-Gal*p*	Tr.	Tr.	Tr.	Tr.	Tr.	Tr.	Tr.	Tr.	Tr.	Tr.	Tr.	Tr.	Tr.	Tr.	Tr.	Tr.	Tr.	Tr.
3-Gal*p*	0.9	Tr.	Tr.	0.5	Tr.	Tr.	Tr.	0.8	0.5	0.7	0.6	Tr.	Tr.	0.6	Tr.	Tr.	Tr.	Tr.
4-Gal*p*	0.6	Tr.	Tr.	Tr.	Tr.	Tr.	Tr.	0.5	Tr.	0.5	Tr.	Tr.	Tr.	Tr.	Tr.	Tr.	Tr.	Tr.
6-Gal*p*	Tr.	Tr.	Tr.	0.5	Tr.	Tr.	0.5	Tr.	Tr.	0.6	0.6	Tr.	Tr.	Tr.	Tr.	Tr.	Tr.	Tr.
2,6-Gal*p*	Tr.	Tr.	Tr.	Tr.	Tr.	Tr.	Tr.	Tr.	Tr.	Tr.	Tr.	Tr.	Tr.	Tr.	Tr.	Tr.	Tr.	Tr.
3,4-Gal*p*	1.2	Tr.	Tr.	0.5	Tr.	Tr.	0.6	Tr.	Tr.	0.9	0.7	Tr.	Tr.	0.5	Tr.	Tr.	0.5	Tr.
3,6-Gal*p*	0.5	Tr.	Tr.	0.6	0.5	Tr.	0.7	0.7	Tr.	0.9	0.9	0.6	0.7	0.9	Tr.	Tr.	Tr.	Tr.
4,6-Gal*p*	1.8	Tr.	Tr.	Tr.	Tr.	Tr.	Tr.	Tr.	Tr.	Tr.	Tr.	Tr.	Tr.	Tr.	Tr.	Tr.	Tr.	Tr.
2,3,6-Gal*p*	0.9	Tr.	Tr.	Tr.	Tr.	Tr.	Tr.	Tr.	Tr.	Tr.	Tr.	Tr.	Tr.	Tr.	Tr.	Tr.	Tr.	Tr.
2,4,6-Gal*p*	1.4	Tr.	Tr.	Tr.	Tr.	Tr.	Tr.	Tr.	Tr.	Tr.	Tr.	Tr.	Tr.	Tr.	Tr.	Tr.	Tr.	Tr.
3,4,6-Gal*p*	Tr.	Tr.	Tr.	Tr.	Tr.	Tr.	Tr.	Tr.	Tr.	Tr.	Tr.	Tr.	Tr.	Tr.	Tr.	Tr.	Tr.	Tr.
2,3,4,6-Gal*p*	1.2	Tr.	Tr.	Tr.	Tr.	Tr.	0.5	Tr.	Tr.	0.8	0.6	Tr.	Tr.	Tr.	Tr.	Tr.	Tr.	Tr.
2,4-Glc*p*+ 2,4-Gal*p*	0.5	Tr.	Tr.	0.5	Tr.	0.5	0.5	Tr.	Tr.	0.5	0.5	Tr.	Tr.	Tr.	Tr.	Tr.	0.5	Tr.
t-Glc*p*	1.8	0.9	1.4	2	1.9	2.2	1.5	1.8	1.4	2.5	3	2.1	1.2	2.3	1.3	1.4	1.4	1.5
2-Glc*p*	0.7	Tr.	Tr.	Tr.	Tr.	Tr.	Tr.	Tr.	Tr.	0.6	0.6	Tr.	Tr.	Tr.	Tr.	Tr.	Tr.	Tr.
3-Glc*p*	1.2	0.6	0.9	1.1	0.8	0.6	1	1.3	0.8	1.6	1.5	1.2	1.4	1.4	1	Tr.	Tr.	0.7
4-Glc*p*	30	40.4	43.7	40.2	50.2	55	43.8	35.4	47	34.9	38.8	47.4	49.8	44.2	51.4	52.7	44.9	46.6
6-Glc*p*	0.7	Tr.	Tr.	0.5	Tr.	Tr.	0.5	Tr.	Tr.	0.7	0.6	Tr.	Tr.	0.5	Tr.	Tr.	Tr.	Tr.
2,3-Glc*p*	Tr.	Tr.	Tr.	Tr.	Tr.	Tr.	Tr.	Tr.	Tr.	Tr.	Tr.	Tr.	Tr.	Tr.	Tr.	Tr.	Tr.	Tr.
3,4-Glc*p*	0.6	0.5	0.6	0.7	0.6	0.8	0.7	0.5	0.5	0.6	0.6	0.5	0.5	0.5	0.5	0.5	0.8	0.5
3,6-Glc*p*	Tr.	Tr.	Tr.	Tr.	Tr.	Tr.	Tr.	Tr.	Tr.	Tr.	Tr.	Tr.	Tr.	Tr.	Tr.	Tr.	Tr.	Tr.
4,6-Glc*p*	3.7	3.9	3.4	2.2	2.5	2.2	3.4	1.9	2.2	3.2	3.1	2.6	2.3	2.7	2.2	1.8	3.8	2
2,3,6-Glc*p*	Tr.	Tr.	Tr.	Tr.	Tr.	Tr.	Tr.	Tr.	Tr.	Tr.	Tr.	Tr.	Tr.	Tr.	Tr.	Tr.	Tr.	Tr.
2,4,6-Glc*p*	Tr.	Tr.	Tr.	Tr.	Tr.	Tr.	Tr.	Tr.	Tr.	Tr.	Tr.	Tr.	Tr.	Tr.	Tr.	Tr.	Tr.	Tr.
3,4,6-Glc*p*	0.5	Tr.	Tr.	Tr.	Tr.	Tr.	0.5	Tr.	Tr.	0.5	Tr.	Tr.	Tr.	Tr.	Tr.	Tr.	Tr.	Tr.
2,3,4,6-Glc*p*	1.7	1.1	0.5	0.7	0.6	0.7	1.2	Tr.	Tr.	1.3	1.1	0.5	Tr.	0.9	Tr.	Tr.	0.6	Tr.
t-Man*p*	1.6	Tr.	Tr.	0.8	Tr.	Tr.	0.6	0.5	Tr.	1.4	1.4	0.6	Tr.	1	Tr.	Tr.	Tr.	Tr.
3-Man*p*	Tr.	Tr.	Tr.	Tr.	Tr.	Tr.	Tr.	Tr.	Tr.	Tr.	Tr.	Tr.	Tr.	Tr.	Tr.	Tr.	Tr.	Tr.

4-Man*p*	0.8	Tr.	Tr.	Tr.	Tr.	Tr.	0.5	Tr.	Tr.	0.7	0.7	Tr.	Tr.	0.6	Tr.	Tr.	Tr.	Tr.
6-Man*p*	Tr.	Tr.	Tr.	Tr.	Tr.	Tr.	Tr.	Tr.	Tr.	Tr.	Tr.	Tr.	Tr.	Tr.	Tr.	Tr.	Tr.	Tr.
2,4-Man*p*	Tr.	Tr.	Tr.	Tr.	Tr.	Tr.	Tr.	Tr.	Tr.	Tr.	Tr.	Tr.	Tr.	Tr.	Tr.	Tr.	Tr.	Tr.
2,6,Man*p*	Tr.	Tr.	Tr.	Tr.	Tr.	Tr.	Tr.	0.5	Tr.	Tr.	Tr.	0.5	Tr.	Tr.	Tr.	Tr.	Tr.	Tr.
3,4-Man*p*	Tr.	Tr.	Tr.	Tr.	Tr.	Tr.	Tr.	Tr.	Tr.	Tr.	Tr.	Tr.	Tr.	Tr.	Tr.	Tr.	Tr.	Tr.
3,6-Man*p*	Tr.	Tr.	Tr.	Tr.	Tr.	Tr.	Tr.	Tr.	Tr.	Tr.	Tr.	Tr.	Tr.	Tr.	Tr.	Tr.	Tr.	Tr.
4,6-Man*p*	Tr.	Tr.	Tr.	Tr.	Tr.	Tr.	Tr.	Tr.	Tr.	Tr.	Tr.	Tr.	Tr.	Tr.	Tr.	Tr.	Tr.	Tr.
2,3,6-Man*p*	Tr.	Tr.	Tr.	Tr.	Tr.	Tr.	Tr.	Tr.	Tr.	Tr.	Tr.	Tr.	Tr.	Tr.	Tr.	Tr.	Tr.	Tr.
2,4,6-Man*p*	Tr.	Tr.	Tr.	Tr.	Tr.	Tr.	Tr.	Tr.	Tr.	Tr.	Tr.	Tr.	Tr.	Tr.	Tr.	Tr.	Tr.	Tr.
3,4,6-Man*p*	Tr.	Tr.	Tr.	Tr.	Tr.	Tr.	Tr.	Tr.	Tr.	Tr.	Tr.	Tr.	Tr.	Tr.	Tr.	Tr.	Tr.	Tr.
2,3,4,6-Man*p*	0.9	Tr.	Tr.	Tr.	Tr.	Tr.	Tr.	Tr.	Tr.	0.5	Tr.	Tr.	Tr.	Tr.	Tr.	Tr.	Tr.	Tr.
t-Rha*p*	Tr.	Tr.	Tr.	Tr.	Tr.	Tr.	Tr.	Tr.	Tr.	Tr.	Tr.	Tr.	Tr.	Tr.	Tr.	Tr.	Tr.	Tr.
2-Rha*f*	Tr.	Tr.	Tr.	Tr.	Tr.	Tr.	Tr.	Tr.	Tr.	Tr.	Tr.	Tr.	Tr.	Tr.	Tr.	Tr.	Tr.	Tr.
4-Rha*p*	Tr.	Tr.	Tr.	Tr.	Tr.	Tr.	Tr.	Tr.	Tr.	Tr.	Tr.	Tr.	Tr.	Tr.	Tr.	Tr.	Tr.	Tr.
2,3-Rha*p*	Tr.	Tr.	Tr.	Tr.	Tr.	Tr.	Tr.	Tr.	Tr.	Tr.	Tr.	Tr.	Tr.	Tr.	Tr.	Tr.	Tr.	Tr.
2,4-Rha*p*	0.5	Tr.	Tr.	Tr.	Tr.	Tr.	Tr.	Tr.	Tr.	0.6	0.6	Tr.	0.5	0.6	Tr.	Tr.	Tr.	Tr.
3,4-Rha*p*	Tr.	Tr.	Tr.	Tr.	Tr.	Tr.	Tr.	Tr.	Tr.	Tr.	Tr.	Tr.	Tr.	Tr.	Tr.	Tr.	Tr.	Tr.
2,3,4-Rha*p*	Tr.	Tr.	Tr.	Tr.	Tr.	Tr.	Tr.	Tr.	Tr.	Tr.	Tr.	Tr.	Tr.	Tr.	Tr.	Tr.	Tr.	Tr.
t-Xyl*p*	1.8	0.9	0.9	1.5	1	0.7	1.2	0.7	1	1.7	1.1	1.2	1.2	0.9	0.9	0.9	1	1.1
2-Xyl*p*	1.3	2	1.8	2.2	1.8	1.5	1.6	1.2	1.7	1.8	1.8	1.9	1.9	1.5	1.9	2.1	1.9	1.9
4-Xyl*p*	7.9	19.2	15.3	16.8	15.9	14	11.9	10.1	15.8	10.3	11.3	13.9	15	12.3	17.2	20	17.3	17.8
2,4-Xyl*p*	1.1	1.9	1.9	1.4	1.5	1.2	1.3	1	1.3	1.6	1.6	1.7	1.6	1.3	1.6	1.4	2	1.6
3,4-Xyl*p*	6.8	6.3	7.2	6.1	6.2	5.9	6	5.4	6.8	7.1	6.3	6.3	6.6	6	6.1	6.1	7.6	6.8
2,3,4-Xyl*p*	2.9	9.4	3.5	1	1.2	0.8	2.5	1.2	1.2	1.9	2	1.3	1.1	1.4	1.3	0.5	3.3	0.9
t-GalA*p*	0.7	Tr.	0.5	0.5	Tr.	Tr.	Tr.	1.7	0.9	0.6	Tr.	Tr.	Tr.	Tr.	Tr.	Tr.	Tr.	0.9
2-GalA*p*	Tr.	Tr.	Tr.	Tr.	Tr.	Tr.	Tr.	Tr.	Tr.	Tr.	Tr.	Tr.	Tr.	Tr.	Tr.	Tr.	Tr.	Tr.
3-GalA*p*	Tr.	Tr.	Tr.	Tr.	Tr.	Tr.	Tr.	Tr.	Tr.	Tr.	Tr.	Tr.	Tr.	Tr.	Tr.	Tr.	Tr.	Tr.
4-GalA*p*	2.2	Tr.	2.3	0.6	0.7	0.6	0.6	13.4	4.9	0.9	1	Tr.	1.1	1.9	0.8	0.6	0.7	3.5
3,4-GalA*p*	Tr.	Tr.	Tr.	Tr.	Tr.	Tr.	Tr.	0.7	Tr.	Tr.	Tr.	Tr.	Tr.	Tr.	Tr.	Tr.	Tr.	Tr.

2,4-GlcA*p*+ 2,4-GalA*p*	Tr.	Tr.	Tr.	Tr.	Tr.	Tr.	Tr.	Tr.	Tr.	Tr.	Tr.	Tr.	Tr.	Tr.	Tr.	Tr.	Tr.	Tr.
t-GlcA*p*	1.8	1.3	1.9	2.2	1.8	1.8	1.8	6	2.5	2.2	2	1.8	1.6	2	1.9	1.5	1.3	2.2
2-GlcA*p*	Tr.	Tr.	Tr.	Tr.	Tr.	Tr.	Tr.	Tr.	Tr.	Tr.	Tr.	Tr.	Tr.	Tr.	Tr.	Tr.	Tr.	Tr.
3-GlcA*p*	Tr.	Tr.	Tr.	Tr.	Tr.	Tr.	Tr.	Tr.	Tr.	Tr.	Tr.	Tr.	Tr.	Tr.	Tr.	Tr.	Tr.	Tr.

Note: Two separate experiments were conducted for each sample.

Table S5 Total carbohydrate quantification of soil obtained from greenhouse (GH) and field (F) through colorimetric analysis.

Sample	Bio Rep	Tech Rep	mg of carbohydrate/ 100 mg of soil	St. Dev
GH	G1	1/2	11.6	0.1
		2/2	11.0	0.1
	G2	1/2	8.6	0.2
		2/2	10.1	1.9
	G3	1/2	12.5	0.6
		2/2	12.5	0.9
	G4	1/2	8.5	0.4
		2/2	11.4	0.1
	G5	1/2	10.0	1.3
		2/2	8.5	0.2
	G6	1/2	9.0	0.4
		2/2	6.9	1.9
F	F1	1/2	9.2	3.1
		2/2	7.2	0.4
	F2	1/2	5.1	0.7
		2/2	4.1	1.3
	F3	1/2	9.6	2.3
		2/2	8.9	1.9
	F4	1/2	6.2	0.4
		2/2	5.9	0.1
	F5	1/2	4.3	1.1
		2/2	5.1	1.0
	F6	1/2	8.4	3.2
		2/2	6.9	3.5

Note: Two separate experiments were conducted for each sample.

Table S6 Daily precipitation recorded at the nearest weather station (Pincher Creek) to our sampling location for 40 days prior to collection in 2022. Amount of total rainfall was 18.4 mm indicating limited soil moisture availability prior to sample collection.

**Date**	**Precipitation (mm)**	**Date**	**Precipitation (mm)**
01-Aug	0	23-Aug	
02-Aug	0	24-Aug	5.3
03-Aug	0	25-Aug	2.2
04-Aug	0	26-Aug	0
05-Aug	0	27-Aug	0.9
06-Aug	0	28-Aug	0
07-Aug	0	29-Aug	0
08-Aug	0	30-Aug	0
09-Aug	0	31-Aug	0
10-Aug	0.6	01-Sep	0
11-Aug	0	02-Sep	0
12-Aug	0	03-Sep	
13-Aug	0	04-Sep	0
14-Aug	0	05-Sep	0
15-Aug	0	06-Sep	0
16-Aug	0	07-Sep	0
17-Aug	0	08-Sep	1
18-Aug	0	09-Sep	8.4
19-Aug	0		
20-Aug	0	**Total**	**18.4**

1.1 Supplementary Figures

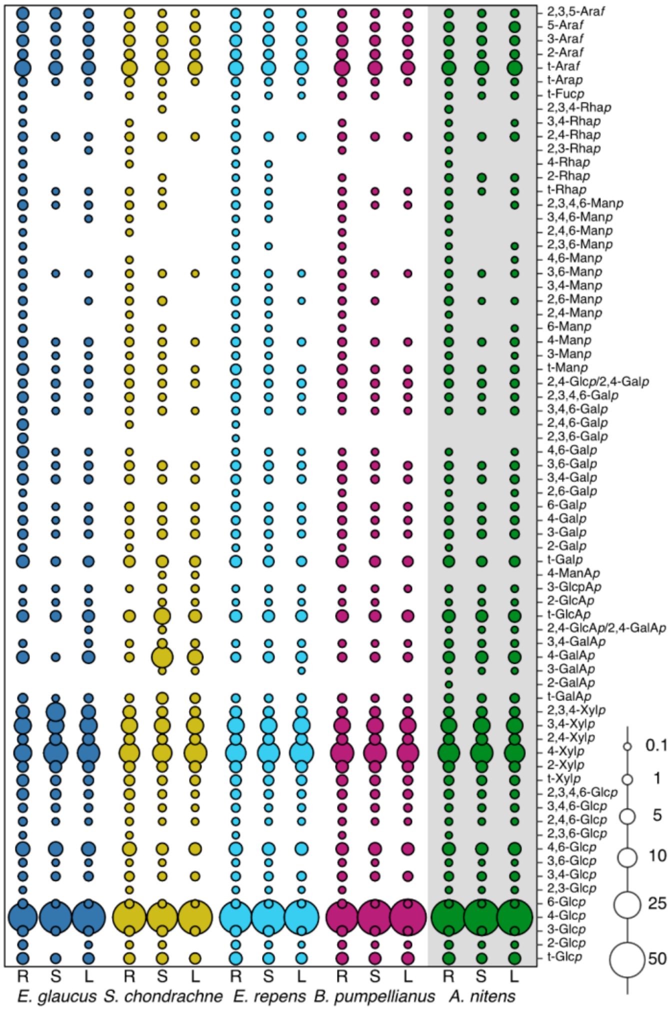

Figure S1 Bubble plot of the different glycosidic linkages (relative abundance) present within the rhizome, stem, and leaf sections of greenhouse-grown Sweetgrass and other Poaceae grass species. Analyzed grass species include *Elymus glaucus* (dark blue), *Setaria chondrachne* (yellow), *Elymus repens* (light blue), and *Bromus pumpellianus* (fuchsia) collected from the field sampling site, and *Anthoxanthum nitens* (Sweetgrass; green) collected from the greenhouse.
